# Inferring the Dynamics of Diversification: A Coalescent Approach

**DOI:** 10.1371/journal.pbio.1000493

**Published:** 2010-09-28

**Authors:** Hélène Morlon, Matthew D. Potts, Joshua B. Plotkin

**Affiliations:** 1 Department of Biology, University of Pennsylvania, Philadelphia, Pennsylvania, United States of America; 2 Department of Environmental Science, Policy, and Management, University of California Berkeley, Berkeley, California, United States of America; University of Oxford, United Kingdom

## Abstract

Recent analyses of the fossil record and molecular phylogenies suggest that there are fundamental limits to biodiversity, possibly arising from constraints in the availability of space, resources, or ecological niches. Under this hypothesis, speciation rates decay over time and biodiversity eventually saturates, with new species emerging only when others are driven to extinction. This view of macro-evolution contradicts an alternative hypothesis that biodiversity is unbounded, with species ever accumulating as they find new niches to occupy. These contrasting theories of biodiversity dynamics yield fundamentally different explanations for the disparity in species richness across taxa and regions. Here, we test whether speciation rates have decayed or remained constant over time, and whether biodiversity is saturated or still expanding. We first derive a general likelihood expression for internode distances in a phylogeny, based on the well-known coalescent process from population genetics. This expression accounts for either time-constant or time-variable rates, time-constant or time-variable diversity, and completely or incompletely sampled phylogenies. We then compare the performance of different diversification scenarios in explaining a set of 289 phylogenies representing amphibians, arthropods, birds, mammals, mollusks, and flowering plants. Our results indicate that speciation rates typically decay over time, but that diversity is still expanding at present. The evidence for expanding-diversity models suggests that an upper limit to biodiversity has not yet been reached, or that no such limit exists.

## Abstract 2

A novel approach to infer diversification dynamics shows that biodiversity is still expanding but at a slower rate than in the past.

## Author Summary

Is species diversity in equilibrium, or is it still expanding? Are there ecological limits on diversity, or will evolution always find new niches for further specialization? These are all long-standing questions about the dynamics of macro-evolution, which have been examined using the fossil record and, more recently, molecular phylogenies. Understanding these long-term dynamics is central to our knowledge of how species diversify and ultimately what controls present-day biodiversity across groups and regions. We have developed a novel approach to infer diversification dynamics from the phylogenies of present-day species. Applying our approach to a diverse set of empirical phylogenies, we demonstrate that speciation rates have decayed over time, suggesting ecological constraints to diversification. Nonetheless, we find that diversity is still expanding at present, suggesting either that these ecological constraints do not impose an upper limit to diversity or that this upper limit has not yet been reached.

## Introduction

Two hypotheses about the dynamics of species diversity prevail in the literature [1]–[3]. According to the first hypothesis, diversity expands without limit. Under this view, the present-day richness of a clade results from a combination of the age of the clade and the speed at which species were generated (i.e., the net diversification rate: speciation rate minus extinction rate) [4]. According to the second hypothesis, evolutionary radiations occur when new ecospaces or resources become available; between such radiations, speciation rates decay and biodiversity saturates [5]–[7]. Under this hypothesis, the variation in standing diversity across clades results from ecological factors such as the amount of space available to species [8],[9], the number of niches they can occupy [10], or the quantity of resources [11],[12] or individuals [13] they partition.

Long-term diversity dynamics have been the subject of long-standing debate. Early work expounded the view that diversity accumulates without limit [14]. Subsequently, Raup [15] and Sepkoski [16] suggested that fossil data are consistent with a logistic model in which diversity is bounded. This debate has continued, mostly nourished by analyses of the fossil record [2],[17],[18]. More recently, molecular phylogenies have provided an alternative source of data, fostering the development of birth–death models of cladogenesis [19],[20]. Hey [21] first compared the performance of models with constant and expanding diversity to reproduce empirical phylogenies, finding more support for the expanding-diversity model. His analyses, however, did not allow rates to vary over time. Further explorations of Hey's constant-diversity model have been surprisingly scarce (but see [22]). Instead, phylogenies have primarily been analyzed in a framework in which diversity increases from a single species at the time of the most recent common ancestor (an assumption made, e.g., by the Yule process). This approach ignores the fact that the ancestor was likely interacting with other species (with no descendants at present), and that diversity might have even remained constant through time ([1], but see [23],[24]). As a consequence, the hypothesis that diversity is constant versus expanding has seldom been tested using molecular phylogenies.

Many studies have examined the hypothesis that rates vary over time, and more particularly that speciation rates decay over time, using at least three different approaches. One approach is based on a summary statistic, gamma, that quantifies the position of nodes in a phylogeny compared to the pure-birth Yule model [25]. Phylogenies with negative gamma values indicate nodes situated towards the root of phylogenies, and have been interpreted as a signature of a slowdown in speciation rates. Although such phylogenies are abundant in nature [5],[6],[25],[26], the interpretation of negative gamma values is controversial [26]. Moreover, the gamma statistic fails to detect slowdowns in speciation rates in the presence of extinction [27], and it is not well suited for comparing the performance of various models or for estimating rates (but see [22]). A second approach compares the likelihood of internode distances under various models of cladogenesis [20],[21],[27]–[30]. This approach offers two advantages: it allows for comparison between different models and for estimation of rates. Applied to empirical phylogenies, such analyses have suggested a decay in the speciation rate over time [27],[30]. However, the levels of extinction estimated by this method are too low to be realistic, suggesting that a major component of diversification is still missing from the modeling [22],[26]. Finally, Venditti et al. [31] recently proposed a third approach, based on the distribution of phylogenetic branch-lengths (distances between ancestor and descendant nodes) rather than the likelihood of internode distances (waiting times between successive nodes). Applying their approach to a large set of molecular phylogenies, the authors concluded that speciation occurs at a constant rate in most taxa.

To summarize the literature discussed above, despite decades of research aimed at investigating the tempo of evolution from molecular phylogenies, three main questions remain unresolved [2],[3],[32]: Is diversity presently saturated, or is it still expanding? Have rates of diversification slowed down over time? Do extinctions leave a detectable signal in empirical phylogenies?

Here, we tackle these questions using a novel approach, inspired by the well-known coalescent process of population genetics [33]. The coalescent process describes the genealogy of individuals sampled from a population “backwards in time,” i.e., from the present to the past. Even though it was originally developed to describe genealogies over short time scales, the coalescent process can also be used to model species' phylogenies—starting from extant species and going backwards in time, back to the time of the most recent common ancestor. The first advantage of this approach to studying cladogenesis is that diversity is not assumed to consist of a single species at the time of the common ancestor. Rather, diversity can take any value at any point in time, including constant diversity through time. The second advantage of the approach is that it easily accommodates incompletely sampled phylogenies, since coalescent theory is by nature a theory of samples. This advantage is of great practical utility, because many phylogenies omit a large proportion of extant species, particularly in species-rich taxa. Finally, the approach also allows comparison of models in which extinction is a free parameter (e.g., the constant-rate birth–death model) to models in which extinction is assumed to be prevalent (e.g., the Hey model; see also [22]); such a comparison allows us to query whether extinction can be detected from molecular phylogenies.

Adapting known results for coalescent times in a population with deterministically varying size [34],[35], we derived a general expression for the likelihood of internode distances in the phylogeny of species sampled at present. We used this expression to approximate likelihoods of internode distances under a variety of birth–death models with time-constant or time-varying diversity, time-constant or time-varying rates, and present or absent extinction. Armed with this theoretical framework, we analyzed empirical phylogenies to investigate whether diversity is expanding or constant, whether rates are time-constant or time-variable, and whether extinctions can be detected in molecular phylogenies. We used two sets of empirical phylogenies: a relatively homogeneous set of phylogenies of birds, with high confidence in branch-length estimates, assembled by Phillimore and Price [6]; and McPeek's broad compilation of phylogenies, which includes chordates, arthropods, mollusks, and magnoliophytes [26]. We analyzed a total of 289 phylogenies.

### Nine Diversification Scenarios

**Figure 1:**
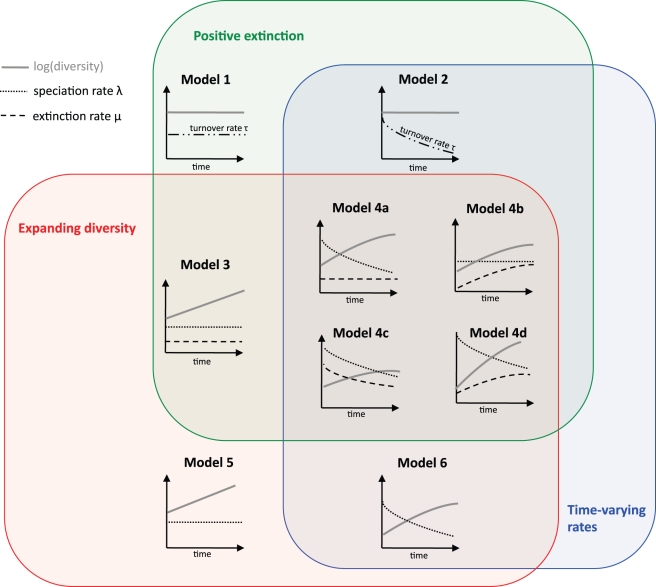
Models of diversification. Schematic illustration of the nine diversification models considered in our analyses. The models can be classified according to three broad criteria: diversity is either expanding over time (in red, Models 3–6) or saturated (Models 1 and 2); rates either vary over time (in blue, Models 2, 4a–4d, and 6) or they are constant over time (Models 1, 3, and 5); and extinctions are either present (in green, Models 1–4) or absent (Models 5 and 6). There are four flavors of models that exhibit expanding diversity with time-varying rates and positive extinction: the speciation rate (λ) varies over time while the extinction rate (μ) is constant (Model 4a); the extinction rate varies over time while the speciation rate is constant (Model 4b); both rates vary over time with a constant extinction fraction (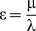; Model 4c); and both rates vary independently over time (Model 4d). When they vary, rates either decay or grow exponentially. The parameters of each model are shown in Table 1.

We considered nine diversification scenarios, illustrated in Figure 1 (see also Table 1). In each of these scenarios, every lineage is equally likely to diversify or go extinct.

**Table 1:** Nine diversification models, their parameters, and empirical support.

Model	Number of Parameters	Model Properties	Parameters		Equation(s) for Rate Variation over Time	Phylogenies for Which Model Is the Best
**Model 1 ** ***(Hey/Moran)***	1	saturated diversity; time-constant rates; positive extinction	τ_0_	turnover rate	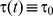	15 (5.2%)
**Model 2**	2	saturated diversity; time-varying rates; positive extinction	τ_0_	turnover rate at present	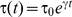	51 (17.6%)
			γ	exponential variation in turnover rate		
**Model 3 ** ***(homogeneous birth–death)***	2	expanding diversity; time-constant rates; positive extinction	λ_0_	speciation rate	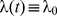	5 (1.7%)
			μ_0_	extinction rate	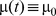	
**Model 4a**	3	expanding diversity; time-varying rates; positive extinction	λ_0_	speciation rate at present	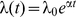	15 (5.2%)
			α	exponential variation in speciation rate		
			μ_0_	extinction rate	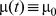	
**Model 4b**	3	expanding diversity; time-varying rates; positive extinction	λ_0_	speciation rate	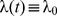	0 (0%)
			μ_0_	extinction rate at present	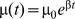	
			β	exponential variation in extinction rate		
**Model 4c**	3	expanding diversity; time-varying rates; positive extinction	λ_0_	speciation rate at present	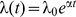	4 (1.4%)
			α	exponential variation in speciation rate		
			ε	extinction fraction	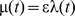	
**Model 4d**	4	expanding diversity; time-varying rates; positive extinction	λ_0_	speciation rate at present	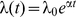	10 (3.5%)
			α	exponential variation in speciation rate		
			μ_0_	extinction rate at present	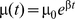	
			β	exponential variation in extinction rate		
**Model 5** ***(Yule)***	1	expanding diversity; time-constant rates; no extinction	λ_0_	speciation rate	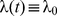	87 (30.1%)
**Model 6**	2	expanding diversity; time-varying rates; no extinction	λ_0_	speciation rate at present	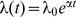	102 (35.3%)
			α	exponential variation in speciation rate		

The final column indicates, for each model, the number and percentage of the 289 empirical phylogenies for which the model exhibits the lowest AICc.

Two of the scenarios (Models 1 and 2) correspond to the hypothesis that diversity is saturated. Species go extinct stochastically and each extinction event is immediately followed by a speciation event, so that diversity remains constant through time. The particular case when the turnover rate (i.e., the rate of events in which an emerging species replaces a species going extinct) is constant through time (Model 1) is identical to Hey's model [21]. Hey's model is itself equivalent to the Moran process of population genetics, which describes the dynamics of individuals as opposed to species. Hey [21] showed that the terminal branches of phylogenies generated under his model are too short to be realistic, yet generalizations of the model to the case in which the turnover rate decays over time (Model 2) may provide a better description of empirical phylogenies (e.g., [22]). Such a decay in rates is expected if species become better adapted over the course of evolution.

The remaining scenarios (Models 3–6) correspond to non-saturated diversity, and they feature independent speciation and extinction events. The model with time-constant speciation and extinction rates (Model 3) is the classical constant-rate birth–death model of cladogenesis [20], which reduces to the Yule process in the absence of extinction (Model 5). The other models (Models 4a–4d and 6) include temporal variation in speciation and/or extinction rates [27],[28],[30]. Rates were assumed to vary exponentially through time, but generalization to any form of time variation is straightforward.

The nine diversification scenarios we consider here represent the range of qualitative cladogenesis processes typically discussed in the cladogenesis literature [1],[19],[20],[27]. These models can be divided into pairs of subsets corresponding to our competing hypotheses for diversity dynamics (Figure 1): models with expanding diversity (in red) versus models with saturated diversity; models with time-varying rates (in blue) versus models with time-constant rates; and models where extinction is present (green) versus models where extinction is absent.

**Figure 2:**
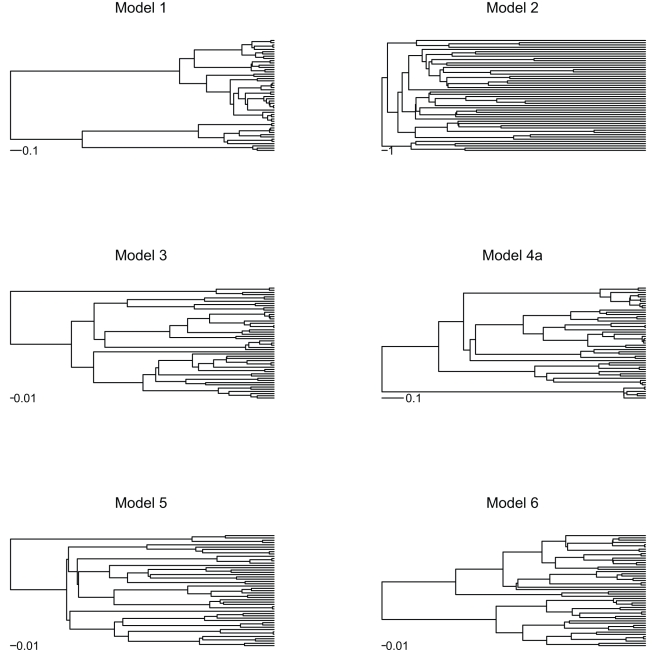
Example phylogenies resulting from different diversification models. Phylogenies simulated under a model with saturated diversity and a constant turnover rate (Model 1) have short terminal branches compared to phylogenies simulated under the pure-birth process (Yule model; Model 5). With saturated diversity but decaying turnover rates, terminal branches become longer (Model 2). Compared to the pure-birth process (Model 5), the presence of extinction pushes phylogenetic nodes towards the tips (Model 3), whereas a decay in speciation rate pushes them towards the root (Model 6). In the presence of both extinction and a decay in speciation rate (Model 4), however, these two effects counteract, producing a phylogeny that appears similar to the pure-birth model. All phylogenies were simulated with the same initial speciation rate (six speciation events per time unit). The extinction rate in Models 3 and 4a was identical (three speciation events per time unit). The exponential variation in speciation rate in Models 2, 4a, and 6 was identical (0.25 per time unit). Note the different time scales.

Phylogenetic trees resulting from these various diversification scenarios have distinct branch-length patterns (Figure 2). Some models produce phylogenies that can easily be distinguished from each other “by eye,” but others produce trees that appear similar and that can be distinguished only through quantitative statistics.

In all nine models, the speciation rate is assumed greater than or equal to the extinction rate at all times. To our knowledge, all models in the cladogenesis literature for which likelihood expressions are available also make this assumption. In nature, however, there is evidence that some clades have lost diversity towards the present, suggesting that extinction events are sometimes more frequent than speciation events [32]. Our coalescent likelihood expression can be used to investigate a scenario with decreasing diversity by assuming an instantaneous mass extinction event in the history of a clade. However, further work remains before the coalescent approach can accommodate general patterns of decreasing diversity (see Materials and Methods).

## Results

### Likelihood of Internode Distances

Consider a clade with 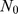 species at the present time, which has evolved according to one of the nine diversification scenarios illustrated in Figure 1. We denote by 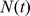 the expected number of species at time 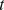 in the past, given the model of diversification and its corresponding parameters (e.g., 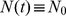 under Models 1 and 2, and 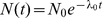 under Model 5). We denote by 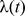 the speciation rate at time 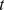 in the past (under Model 1 and 2, 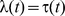, where 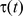 is the turnover rate at time 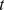 in the past). We consider a phylogeny of 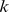 species randomly sampled in the clade at the present time. This phylogeny has 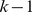 internal nodes, and 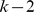 internode distances. The distance between node 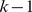 and the present is excluded because it does not correspond to a waiting time between cladogenesis events.

Adapting results known for the Kingman's coalescent with deterministically varying population size [34], the log-likelihood 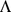 of the distances 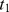, 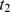, …, 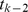 between nodes in the phylogeny (nodes are numbered from the root to the tips, and 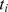 is the time-length between node 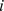 and node 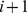) is given by
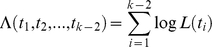
with
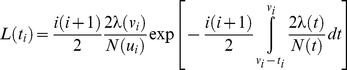
(1)where 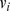 is the time-length between node 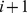 and the present (see Materials and Methods). This expression is valid only under the assumption that 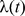 is greater than or equal to 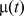 (see Materials and Methods). Furthermore, the stochastic number of species present at time *t* has been approximated by its deterministic expectation, N(*t*). This approximation is critical to our analytical approach, as it makes the corresponding coalescent process tractable. We show below that this approximation is accurate over a broad range of parameters.

The general expression above can be used to derive an approximate likelihood for the internode distances under each of the nine diversification scenarios illustrated in Figure 1 (Appendix S1 in Text S1). Given an empirical phylogeny, these expressions can then be used to estimate rates (by maximum likelihood), or to compare the performance of various models. For example, the likelihood of 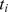 under the simple Hey model (Model 1) is
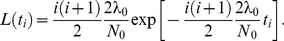
(2)This equation shows that it is not possible to estimate the speciation rate and the number of species at present independently, given that the equation involves only the ratio of the two parameters. Therefore, we assume that clade size at present is known. In typical applications, accurate estimates of clade size exist for most groups.

### Robustness of the Coalescent Approach

**Figure 3:**
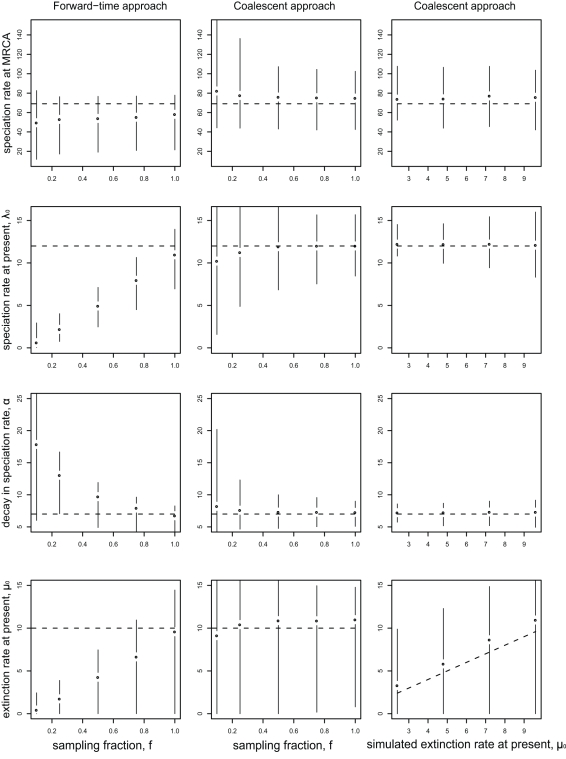
The coalescent method provides robust estimates of diversification rates from incompletely sampled phylogenies. The figure shows maximum likelihood parameter estimates for phylogenies simulated under Model 4a (extinction rate is constant over time and speciation rate decays exponentially). The true, simulated parameters of diversification are indicated by dashed lines (expressed in number of events per time unit). Points and error bars indicate the median and 95% quantile range of the maximum likelihood parameter estimates, across 1,000 simulated phylogenies for each parameter set. The right column shows the estimated extinction rate at present, compared to its true, simulated value. Before estimating parameters, species were randomly sampled from the simulated phylogenies. In the left and middle columns the sampling fraction *f* ranged from 10% of species (poorly sampled) to 100% of species (fully sampled). In the right column, *f* = 75% of species were sampled. MRCA, time at the most recent common ancestor.

Using simulations, we tested the ability of the coalescent approach to determine the properties of the true, underlying cladogenesis process, from complete and incomplete phylogenies (Figures 3 and S1 [in Text S1]). We found that the approach performed well with either complete or incomplete taxa sampling, and under both hypotheses of expanding (Figure 3) or constant (Figure S1 in Text S1) diversity. The method also performed well in the presence of low and high levels of extinction (Figure 3).

Under the scenario with expanding diversity, a forward-time approach (i.e., an approach in which the process of cladogenesis is considered from the past to the present, as opposed to the backwards-time coalescent approach) exists for estimating rates [27],[28],[30]. The forward-time approach has the advantage over ours that it does not require approximating diversity with a deterministic expectation, and it thus may be more accurate. However, we did not find a striking difference in the performance of the two methods. Although extinction rates were slightly overestimated with the coalescent approach, this bias was small in comparison with the large variability around expected values obtained with either the coalescent or the forward-time method (Figure 3).

The forward-time approach commonly used in the literature does not simultaneously accommodate both time-varying rates and incomplete sampling of extant species (although this could in principle be accommodated; see [28],[36],[37] and Discussion). It is well recognized that this approach should, in principle, only be used on completely sampled phylogenies. However, many empirical phylogenies omit a large proportion of extant species, and little is known about the accuracy of forward-time methods when applied to incomplete phylogenies. Our analyses indicate that such methods will produce strongly biased estimates, even when as many as 75% of extant species are present in the phylogeny. For example, in the case of the model with decaying speciation rate and constant extinction rate (Model 4a), incomplete sampling leads to an underestimation of the speciation rate at the time of the most recent common ancestor, an overestimation of the decay in speciation rate, and an underestimation of the extinction rate (Figure 3). By comparison, the coalescent approach produced accurate estimates of rates when as few as 10% of the extant species were sampled. Although informative, this comparison is not entirely fair, because the coalescent approach is designed to describe the genealogy of samples, unlike the commonly used forward-time approaches (see also [38]).

Our simulations also show that the coalescent approach accurately identifies whether the underlying cladogenesis process is saturated or not, even under incomplete sampling. For example, out of 100 phylogenies simulated under a model with saturated diversity and constant turnover (Model 1, N_0_ = 100), sampled with a fraction *f* = 0.75, 83 were best fit (i.e., had the lowest second-order Akaike's Information Criterion [AIC*_c_*]; see Materials and Methods) by a model with saturated diversity (69 by Model 1 and 14 by Model 2) and, 78 were best fit by a model with constant rates (69 by Model 1 and nine by Model 3).

The coalescent approach is also able to detect decays in speciation rates. All phylogenies shown in Figure 3—generated by Model 4a, which features positive, constant extinction, and decaying speciation rates—were best fit by a model with decaying speciation rate: Models 4a, 4d, 4c, and 6 were most likely in ∼44%, ∼46%, ∼6%, and ∼4% of the simulated phylogenies, respectively. By comparison, using the approach recently proposed by Venditti et al. [31] (see also Materials and Methods), 69% of phylogenies simulated with a decaying speciation rate were best fit by models in which speciation occurs at a constant rate (i.e., the branch-length distributions of ∼67% and ∼2% of the phylogenies were best fit by the exponential and lognormal distributions, respectively; the remainder were best fit by a Weibull distribution). Thus, the method of Venditti et al. is not well adapted to detecting a decay in speciation rates over absolute time. This comparison does not remove the value of the approach by Venditti et al., which was designed to detect a dependence of speciation rates on the divergence time from an ancestral species rather than on absolute time (see Discussion).

### Diversity Is Expanding with Decaying Rates

We compared the performance of the nine diversification scenarios illustrated in Figure 1 in describing 289 empirical phylogenies (see Materials and Methods). We found that for a large number of phylogenies (102 out of 289; ∼35%; Table 1), the most likely model featured a time-decaying speciation rate and no extinction (Model 6). The pure-birth Yule model was the most likely model in another ∼30% of the phylogenies (Model 5). Finally, the model with saturated diversity and decaying turnover rate (Model 2) was the most likely in ∼18% of the phylogenies. Each of the other models was the most likely in less than 6% of the phylogenies. In particular, the constant-rate birth–death model (Model 3) was the most likely in only five out of 289 phylogenies (less than 2% of the phylogenies).

**Figure 4:**
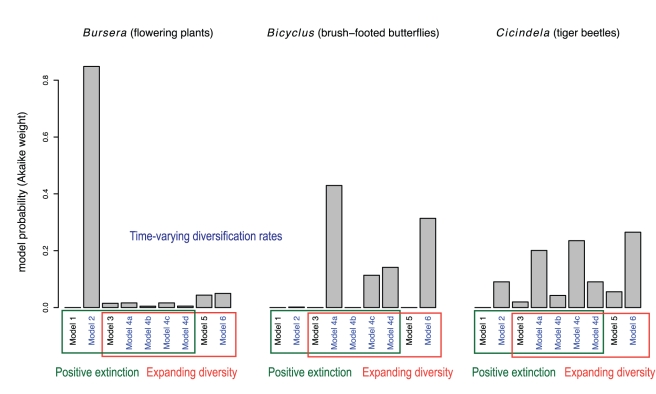
Dynamics of diversification in three empirical phylogenies. Each bar represents the probability—measured as the Akaike weight—that the phylogeny arises from the corresponding model, among the set of nine models considered. The phylogeny of the genus *Bursera*, comprising 73% of known species in that genus, overwhelmingly supports Model 2. Thus, the *Bursera* phylogeny is consistent with the hypotheses that diversity is saturated and that the turnover rate varies over time. The phylogeny of the genus *Bicyclus*, comprising 68% of known species, is consistent with the hypotheses that diversity is expanding and that speciation rates vary. The phylogeny of the genus *Cicindela*, comprising 84% of recognized species, is also largely consistent with the hypotheses that diversity expands and rates vary. However, the dynamics of diversification are less clear-cut in the *Cicindela* phylogeny, because models with saturated diversity and constant rates also have positive probabilities. Although there is high confidence for the presence of extinction in the phylogeny of *Bursera*, models with or without extinction are about equally likely in the phylogenies of *Bicyclus* and *Cicindela*. Models with time-varying diversification rates are written in blue text.

Sometimes the model with the smallest AIC*_c_* score, among a set of candidate models, is not highly supported. This happens, for example, when both the first- and second-best models have similar AIC*_c_* scores. Intuitively, the difficulty in distinguishing between models reflects the fact that different diversification scenarios may result in phylogenies with similar branch-length patterns (Figures 2 and S2 [in Text S1]; see also [19],[31],[32]). To evaluate a model's support among a set of alternatives, we used Akaike weights—a measure of the probability of a given model among a set of candidates ([39]; see Materials and Methods). For a few phylogenies, the single most likely model was highly probable (e.g., phylogeny of the genus *Bursera*; Figure 4). But for many other phylogenies, the most likely model had a less than 50% chance of actually being the true model (e.g., the phylogenies of *Bicyclus* and *Cicindela*; Figure 4).

We assessed three competing hypotheses about diversity dynamics: (1) whether diversity is expanding or constant over time, (2) whether rates vary or are constant over time, and (3) whether extinctions leave a detectable signal in molecular phylogenies. To test each of these questions we used the following procedure. We first selected the model with lowest AIC*_c_* in each subset. For example, to test the hypothesis of expanding versus saturated diversity, we selected for each phylogeny the model with lowest AIC*_c_* among the models with expanding diversity, and the model with lowest AIC*_c_* among the models with saturated diversity. We then evaluated the relative probability of these two models, based on their Akaike weights. The distribution of the relative probabilities across empirical phylogenies serves as a quantitative resolution to each of the three competing hypotheses we set out to test. This selection procedure provides a robust inference method (Figure S3 in Text S1).

**Figure 5:**
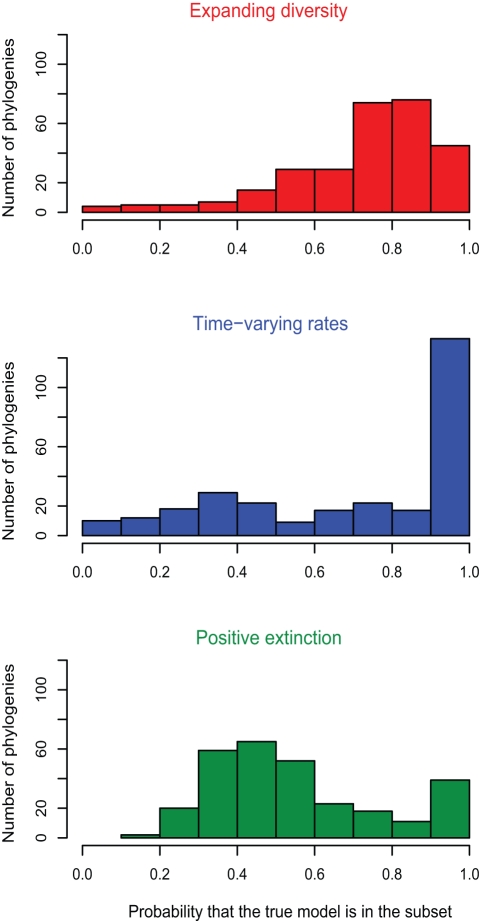
Dynamics of diversification among 289 empirical phylogenies. The red histogram shows, for each of the 289 phylogenies, the relative probability of the best model with expanding diversity versus the best model with saturated diversity. The blue histogram shows the relative probability of the best model with time-varying rates versus the best model with constant rates. The green histogram shows the relative probability of the best model with positive extinction versus the best model without extinction. The relative probabilities of two models are calculated using their Akaike weights. Most empirical phylogenies are consistent with the hypotheses that diversity is expanding (in red) and that speciation rates vary through time (in blue). Extinction is not detected in most phylogenies (in green).

We found that for most phylogenies the best model with expanding diversity was more probable than the best model with saturated diversity (∼77% of the phylogenies; Figure 5). In addition, for most phylogenies the best model with time-varying rates was more likely than the best model with time-constant rates (∼65% of the phylogenies). In particular, we typically found best-fit models that exhibit decaying speciation rates or net diversification rates. Furthermore, the best model without extinction was typically more likely than the best model with extinction (in ∼65% of the phylogenies). These results were consistent across the chordate (including birds), mollusk, arthropod, and magnoliophyte phylogenies, with no striking differences across phyla (Figures S4, S5, S6, S7 in Text S1).

The result that most phylogenies are consistent with expanding diversity and time-varying rates was robust to various tests. First, this result was not an artifact of the coalescent approach or of the model-selection procedure, since a poor fit of models with expanding diversity and time-varying rates was obtained when the procedure was performed on phylogenies simulated under a model with saturated diversity and constant rates (Model 1; Figure S3 in Text S1). Second, this result held when considering only the bird phylogenies from Phillimore and Price [6], suggesting that it was robust to the method of phylogenetic construction (Figure S8 in Text S1). Third, this result was independent of the fraction of species sampled in the phylogenies, confirming the robustness of the coalescent inference to undersampling (Figure S9 in Text S1). Finally, inhomogeneity in diversification rates across lineages within phylogenies could have led to spurious estimates, and potentially to misleading inference [40]. However, the phylogenies we used included a narrow taxonomic range of species, which likely limited rate heterogeneity. Furthermore, we found no dependence between our results and the tree-splitting parameter, a measure of phylogenetic imbalance that reflects heterogeneity in the speed at which lineages diversify (Materials and Methods and Figure S9 in Text S1). This suggests that our observation of expanding diversity with time-decaying rates was not an artifact of inhomogeneous diversification rates.

Our test of time variation in rates was conservative. We found evidence for time variation even though we allowed only exponential variations in rates. Allowing additional forms of temporal variations would, if anything, increase the number of phylogenies for which we infer some form of time variation. Furthermore, we found a positive correlation between the probability of the best model with time-varying rates and clade size (Figure S10 in Text S1), suggesting that small trees, if they were to influence the results, would influence them towards an absence of time variation in rates.

Our test of expanding diversity, however, could be biased by the presence of small trees. Indeed, we found a negative correlation between the probability of the best model with expanding diversity and phylogeny size (Figure S10 in Text S1). However, the support for expanding diversity held even when considering only the phylogenies with more than ten tips (Figure S11 in Text S1) or more than 50 tips (Figure S12 in Text S1), suggesting robust evidence for expanding diversity.

### The Coalescent Approach Can Detect Signatures of Extinction

Although models without extinction were generally more likely than models with extinction, our results suggest that extinctions can sometimes leave a detectable signal in molecular phylogenies. In those phylogenies for which extinction was detected (100 phylogenies, or 35%), the estimated ratio of present-day extinction and speciation rates was very high (mean extinction fraction across phylogenies with positive extinction: 0.94±0.014 [1 standard error]). As a result, the mean extinction fraction at present across all phylogenies was nontrivial (0.32±0.013). We observed a positive correlation between clade size and the probability that the best model features extinction (Figure S10 in Text S1). In addition, for ten of the 16 phylogenies with more than 50 tips, the best model with extinction was more likely than the best model without extinction (Figure S11 in Text S1; see also Appendix S4 in Text S1). This suggests either that species are more often subject to extinctions in big clades or that the failure to detect extinction in many phylogenies is linked to their small size. These results illustrate the potential superiority of the coalescent approach over the forward-time approach for estimating extinction rates from molecular phylogenies (see Discussion).

### Coalescent Models Produce Realistic Gamma Statistics

Our analysis of diversification rates has focused on the best-fit model amongst a set of nine alternative models. But this begs the question: does the best-fit model itself provide a reasonably accurate description of the empirical phylogeny? For example, none of the models accounts for rate variation across lineages. As a result, empirical trees are typically more imbalanced than those predicted by the best-fit model (Figure S14 in Text S1). This is in agreement with previous studies showing that phylogenies arising from birth–death models are more balanced than empirical ones [19].

Nonetheless, we have verified that our best-fit model provides a good fit in at least one important respect: the gamma statistic. The gamma values of the best-fit models accurately reproduce the observed gamma values of the empirical phylogenies (Figure S14 in Text S1), even though our fitting procedure did not explicitly include any information about gamma. Thus, our modeling approach produces phylogenies with realistic branch-length patterns.

## Discussion

The relative importance of ecological interactions and the physical environment in driving macro-evolutionary patterns has been the subject of a long-standing debate. We have developed a coalescent-based approach to study diversity dynamics. Applying this tool across a diverse set of 289 empirical phylogenies, we found that speciation rates tend to decay over time, but that diversity is typically still expanding at present. These results suggest that diversification is the product of bursts of speciation followed by slowdowns in speciation rates as niches are filled, but not yet exhausted.

The coalescent framework developed here is particularly well suited to the study of incomplete phylogenies. This is of practical importance, because fully sampled phylogenies are rarely available. By contrast, time-forward methods cannot easily accommodate missing species, which limits their practical utility. Incomplete sampling of extant species leads to a lengthening of terminal branches, modifying the series of internode distances as well as the distribution of phylogenetic branch-lengths. For example, sampling reduces gamma values, which can lead to a misleading rejection of the constant-rate birth–death model [35]. In this specific case, corrections can be made using Monte Carlo simulations [35]. In the case of phylogeny-based maximum likelihood inference, Nee et al. [28] proposed to treat sampling as a mass extinction event at present (see also [36],[38] and Appendix 2 of [37]). The corresponding likelihood expression, however, was only derived in the case of the constant-rate birth–death model. Our approach provides a more general expression for use with incomplete phylogenies, allowing rates to vary with any functional dependence over time, and clade size to vary or be constant over time.

The coalescent framework can produce phylogenies with a wide range of branch-length patterns, including phylogenies with negative gamma values. In the field of macro-evolution, the coalescent has mostly been discussed in the context of Hey's model [21]. This model corresponds to the coalescent with constant population size and constant generation time, which—applied to a model of cladogenesis—produces phylogenies with short terminal branches and positive gamma values, in disagreement with empirical evidence. Our approach instead allows both population size (i.e., clade size in a macro-evolutionary context) and generation time to vary over time. As we have seen, this more general approach produces phylogenies with realistic branch-length patterns, and realistic gamma values.

The coalescent approach has allowed us to detect extinction in molecular phylogenies. Forward-time approaches typically produce estimates of extinction rates that are far too small to match the fossil record [27]. This discrepancy has motivated the development of models that directly incorporate species interactions [26], or models in which extinction is forced to happen [22]. However, the absence of likelihood expressions for these more complicated models prevents comparison with simpler, more parsimonious models. Using an approach that allows model comparison, we have found that many phylogenies produce low extinction estimates, but others produce high estimates, resulting in significant inferred extinction levels overall. One explanation for the difference between our results and those obtained with the standard forward-time approach is that the coalescent approach allows diversity to take any value at the time of the most recent common ancestor. Hence, clades can reach present-day diversity even with high extinction levels. By contrast, the forward-time approach typically assumes that diversity increases monotonically from a single species to the diversity at present—which may suppress the inferred rate of extinction (but see [23],[24]).

There are several potential extensions and applications of the coalescent approach in macro-evolution. First, our assumption that the speciation rate is always greater than or equal to the extinction rate could be relaxed. This would allow us to consider scenarios in which diversity decays over time, which is biologically relevant and might influence our conclusions. Such scenarios cannot be easily accommodated by the other modeling approaches in the literature [1]. Second, the coalescent framework should allow us to incorporate information from fossil data. For example, if reliable estimates of diversity at one or several points in time were available from fossil data, these estimates could be incorporated into the expression for the likelihood of internode distances, yielding more robust inferences. Finally, by adapting results on the coalescent with spatial structure (see, e.g., [41]), we could test hypotheses about both the temporal and spatial modes of diversification—e.g., when and where did species diversify? All of these extensions remain topics for future research.

There are nevertheless limitations to the coalescent approach. We used an expression for coalescence times derived from population models with deterministically varying size. Using this expression to analyze phylogenies (evolutionary relationship among species, not individuals) required approximating the number of species at a given time (a stochastic variable) by its deterministic expectation. Within the range of parameters we tested, this approximation created only a small bias in the estimation of extinction rates (see, e.g., Figure 3). We do not, however, exclude the possibility that this approximation may bias estimates for other parameter values [38]. We also assumed that species are randomly sampled, whereas they are often sampled with the goal of maximizing phylogenetic breath [26]. The McPeek dataset we analyzed, however, was designed to avoid this bias [26].

Another major limitation of our approach is that we did not account for rate variation across lineages. In other words, within a phylogeny and at any given time, all species were assumed equally likely to diversify, and equally likely to go extinct. This assumption is made by analytical forward-time models as well, but it is strongly violated in nature. Species colonizing new areas or acquiring beneficial traits diversify faster than others [7],[42],[43], and extinctions are clustered on the phylogeny, i.e., species within some clades are more likely to go extinct than others [44]. Consequently, empirical phylogenies are more imbalanced than predicted by models with homogeneous rates [19], and inferences based on models with homogeneous rates might be biased [40]. Extending our approach to account for inhomogeneous rates would require considering coalescent times in a population under selection. Although mathematical solutions to this problem are typically not available, an efficient simulation approach exists (the so-called ancestral selection graph [45]), which could be adapted to model differential diversification rates across lineages.

Alternative approaches for modeling phylogenies with inhomogeneous rates across lineages exist, but they also have limitations. One approach that produces realistic levels of imbalance stems from the neutral theory of biodiversity [13],[46]. Under the neutral theory with point mutation, there is a fixed probability of speciation per individual, so that speciation rates vary across lineages according to population sizes. However, the point-mutation model of speciation is highly disputable [47]. Furthermore, the neutral theory of biodiversity produces phylogenies with terminal branches that are unrealistically short, unless a steep increase in population sizes towards the present is assumed (personal communication, Franck Jabot). Another approach consists of modeling cladogenesis in parallel to the evolution of species' traits, with speciation and extinction rates depending on these traits [37],[48]–[50]. Although powerful, this approach necessitates collecting information on both the phylogenetic relationships of extant species and their traits. Furthermore, such inferences assume a priori that rate variation across species is linked to variations in their traits, as well as which traits influence diversification rates.

Despite the limitations in our modeling, our empirical results strongly suggest that diversification rates vary over time in many taxa (Figure 5). The best model with time-varying rates was more likely than the best model with time-constant rates in 182 of 289 phylogenies (∼63%), and was at least three times more likely in 147 (∼51%) of them.

Our results on the prevalence of decaying speciation rates is apparently at odds with a recent meta-analysis of molecular phylogenies by Venditti et al. [31], who concluded that speciation typically occurs at a constant rate over time. This comparison is somewhat inappropriate, however, because the method of Venditti et al. was designed to detect changes in speciation rates with the age of a taxon, as opposed to with absolute time. As we have demonstrated above using simulated phylogenies, our coalescent technique has greater power to detect changes in speciation rates over absolute time than the approach by Venditti et al. As a result, even though our coalescent method detects decaying speciation rates in the majority of our empirical phylogenies, the method of Venditti et al. would infer time-varying rates (i.e., a Weibull distribution of branch-lengths) in only 17% of the empirical phylogenies analyzed here (data not shown). Given the strong evidence we find for a decay in speciation rate over absolute time using the coalescent approach, which confirms results obtained by other inference techniques [5],[6],[25],[27],[51],[52], we conclude that it might be premature to reject diversification in bursts [5],[6],[27],[52] (adaptive radiations followed by a slowdown in speciation rate) in favor of diversification at a constant pace in response to rare stochastic events [31].

Our analysis also suggests that diversity is not saturated, but rather presently expanding. This hypothesis has been tested previously using fossil data, yielding contradictory results [2],[15],[16],[18]. Using molecular phylogenies, the constant-diversity hypothesis has been tested only under scenarios with time-constant rates [21]. Recently, the resurgence of the idea that diversity is limited by available resources has encouraged the development of models for cladogenesis with saturated diversity [22]. However, our findings suggest that birth–death models with time-varying rates should be favored over the classical constant-rate birth–death model or models with saturated diversity. More generally, our results suggest that an understanding of both evolutionary history (clade age and diversification rates) and ecological constraints (geographical space, resource availability, and competition among species) is necessary to explain present-day diversity and its variation across clades and regions [3].

## Materials and Methods

### Likelihood of Internode Distances

Consider the genealogy of 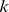 individuals sampled in a population with deterministically varying size, evolving under the Wright-Fisher process (at each generation, all individuals die and are replaced; each offspring selects a parent randomly from the previous generation). We number nodes from the root to the tips, denote 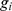 the time-length between node 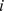 and node 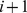, and 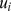 the time-length between node 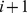 and the present, measured in units of generation time (time for a complete turnover of individuals). Using the standard coalescent approximation, Griffiths and Tavare [34] have shown that the internode distances (e.g., 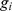) are distributed according to
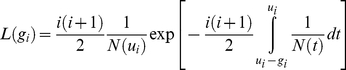
(3)where 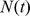 is population size at time 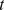 in the past. This result has been widely used in population genetics to infer demographic history using genealogies (e.g., [35],[53]).

By analogy, replacing individuals by species, we used the coalescent approximation to infer diversity dynamics using phylogenies. In the case of an evolving population, the generation time is assumed constant over time. For a clade evolving with varying speciation rates, the generation time (time for a complete turnover of species) varies: intuitively, a complete turnover of species is reached faster when speciation rates are higher. If 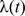 is not less than 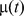 at all times, the generation time at time *t* in the past is given by 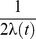, where 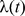 is the speciation rate at time *t* (in real time units). The change of variable (real time units versus generation time units) yields the likelihood expression in the text.

### Simulations

We used forward-time simulations to construct phylogenies under different models of cladogenesis. To simulate a phylogeny of size 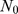 under Models 1 and 2, we started with an artificial phylogeny consisting of 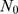 species connected to the root by a polytomy. We simulated the time of each turnover event (i.e., an extinction event immediately followed by a speciation event) using the exponential distribution with rate parameter 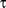 at the time of the previous turnover event (for the first turnover event we used the initial turnover rate 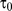). At each event, a lineage picked at random was removed while another lineage, also picked at random, was replaced by two descendant lineages, and the turnover rate's value was updated (in the case of Model 2). The process was simulated until time exceeded a predetermined value. Note that the initial polytomy disappears as soon as all but one of the initial lineages go extinct.

To simulate all other diversification scenarios (Models 3–6), we started with a single lineage and simulated events at rate 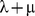 (i.e., speciation plus extinction rates at the time of the previous event; for the first event we used the initial rate 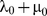). At each event, a lineage picked at random was replaced by two descendant lineages with probability 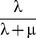, and removed with probability 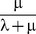, and the speciation and extinction rates were updated (according to the equations in Table 1). The process was simulated until time exceeded a predetermined value.

### Comparison of the Coalescent Approach to Alternative Approaches

To estimate speciation and extinction rates using the forward-time approach (Figure 3), we used maximum likelihood estimation as implemented in the SPVAR model of [27], using the Laser package in R [54].

To evaluate the fits of the various models from Venditti et al. [31] on a given phylogeny, we first obtained the distribution of phylogenetic branch-lengths. Following the authors, we excluded terminal branch-lengths because they do not reflect speciation events. We fitted the exponential, Weibull, lognormal, variable rates, and normal distribution to this distribution of branch-lengths (Table 1 of [31]). All models besides the Weibull correspond to scenarios of diversification in which speciation occurs at a constant rate. We obtained the maximum likelihood parameters of each model using the Nelder-Mead simplex algorithm implemented in R [55]. To measure goodness of fit, we computed the AIC*_c_*:
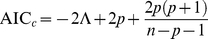
(4)in which 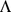 is the log-likelihood of the branch-lengths, 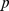 is the number of parameters in the model, and 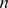 is the number of observations (i.e., the number of branch-lengths) [39].

### Empirical Phylogenies

We used two sets of published phylogenies: 44 from Phillimore and Price [6] and 245 from McPeek [26], for a total of 289 phylogenies (the phylogeny of Estrildidae from Phillimore and Price [6] was not included because it is not publicly available). The phylogenies from Phillimore and Price [6] were exclusively bird phylogenies; they were constructed by the authors from sequence data, using a relaxed-clock Bayesian method implemented in BEAST (see Phillimore and Price [6] for details). This approach yielded a distribution of trees for each clade. We first made sure that our results did not depend on the choice of the tree. Thereafter, we used for each clade a randomly chosen tree from the distribution of trees. Estimates of present-day clade richness were provided by the authors (Table 1 of [6]).

The phylogenies from McPeek [26] included 55 arthropod phylogenies, 140 chordate phylogenies, 11 mollusk phylogenies, and 39 magnoliophyte phylogenies, compiled by the authors from the literature (see [4] and [26] for details). Small phylogenies were included in the compilation, in order to avoid the potential bias associated with analyzing only species-rich clades [1]. The authors also excluded phylogenies where the hypothesis of random sampling was obviously violated (i.e., phylogenies in which sampling was biased in order to maximize the breath of species sampled). Estimates of present-day clade richness were provided by the author. Some phylogenies had polytomies, reflecting nodes were resolution was contentious. Since the order of resolution does not matter in analyses involving only internode distances, we resolved nodes randomly. We assigned arbitrarily small internode distances between them (10^−6^ My), and checked that the results were robust to the arbitrary value chosen.

### Hypothesis Testing

For each phylogeny, maximum likelihood optimization for each of the nine models was performed using the Nelder-Mead simplex algorithm implemented in R [55]. To measure goodness of fit, we computed the AIC*_c_* as described above (Equation 4). Here 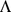 is the log-likelihood of internode distances and *n* is the number of internode distances included in the likelihood calculation, i.e., 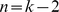, where *k* is the number of tips in each phylogeny (we recall that the time between the last speciation event and the present was omitted since it does not correspond to a waiting time between two speciation events). To evaluate the relative performance of a given model *l* within a set of *R* candidate models, we computed the model's Akaike weight 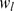 as
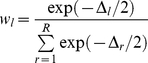
(5)where 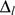 is the difference in AIC*_c_* between model *l* and the best model (i.e., the model with smallest AIC*_c_*). The Akaike weight of a given model may be interpreted as the probability that the model is the true model, given the set of candidate models [39]. The relative probabilities of two models *l* and *k* were then calculated as 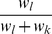 and 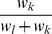.

### Estimating Extinction

To estimate the level of extinction in a given phylogeny, we estimated the level of extinction provided by the best-fit model for this phylogeny. To obtain a measure of extinction comparable across phylogenies, we reported the extinction fraction (extinction rate divided by speciation rate) at present. In the case of Models 1 and 2 (saturated diversity models), the extinction fraction was assigned a value of 1, since each speciation event is directly followed by an extinction event. In the case of Models 5 and 6 (models without extinction), the extinction fraction was assigned a value of 0. The mean extinction fraction was computed both across phylogenies where the best-fit model was a model with extinction and across all phylogenies.

### Summary Statistics

We adopted the broadly used gamma statistic to summarize information on phylogenetic branch-lengths [25]. The gamma statistic follows the standard normal distribution under the pure-birth Yule model, and takes negative values when phylogenetic nodes are closer to the root than expected under the Yule model. To summarize information on phylogenetic imbalance, we used the tree-splitting parameter implemented in the apTreeshape package in R [56]. The tree-splitting parameter is the maximum likelihood estimate of a single-parameter family of split distributions (i.e., probability distributions describing the left sister clade size conditional on the parent clade size) encompassing the split distribution of the Yule model. The expected value of the tree-splitting parameter is zero under the Yule model, negative for trees more imbalanced than expected under the Yule model, and positive for trees more balanced than expected under the Yule model [57],[58]. We chose this measure because, contrary to other measures, its expectation under the Yule model is independent of clade size.

### Performance of the Best-Fit Model

We assessed for each empirical phylogeny how well the best-fit model (with associated best-fit parameters) actually represented the data. We simulated for each empirical clade 100 phylogenies according to the model, as described above. We then randomly sampled species from each simulated phylogeny, with the sampling fraction corresponding to the empirical data. Finally, we compared empirical and simulated phylogenies using a summary statistic that reflects phylogenetic imbalance (the tree-splitting parameter; [57],[58]) and a summary statistic that reflects phylogenetic branch-lengths (the gamma statistic; [25]).

## Supporting Information

Text S1 **Supplementary Data.** Contains Figures S1, S2, S3, S4, S5, S6, S7, S8, S9, S10, S11, S12, S13, Table S1, and Appendices S1, S2, S3, S4.
(3.27 MB PDF)

## Abbreviations

AIC*_c_*: second-order Akaike's Information Criterion
