# Supplementary material for: Inferring the Dynamics of Diversification: A Coalescent Approach
Source: PLoS Biol. 2010 Sep 28;8(9):e1000493. doi: 10.1371/journal.pbio.1000493 (PMC2946937; doi:10.1371/journal.pbio.1000493)
Supplement: Text S1 — Supplementary Data. Contains Figures S1, S2, S3, S4, S5, S6, S7, S8, S9, S10, S11, S12, S13, Table S1, and Appendices S1, S2, S3, S4. (3.27 MB PDF) [file pbio.1000493.s001.pdf]

Ecological Limits on Diversification

Supplementary Information

Hélène Morlon[1], Matthew D. Potts[2], Joshua B. Plotkin[1]

1. Department of Biology, University of Pennsylvania, Philadelphia, PA, USA
2. ESPM, University of California, Berkeley, CA, USA

Appendix 1. Likelihood of internodes distances.

Using the general expression for the likelihood of internode distances under deterministically varying clade size (Equation 1 from the main document), the likelihood of internode distances can be derived under a variety of diversification scenarios. This entails deriving the expected number of species $N(t)$ at any given time, given the number of extant species N_0 and the underlying scenario of diversification. We recall that time is measured from the present to the past.

Under models with constant diversity (e.g. Models 1 & 2), $N(t) = N_0$ at all times, and the speciation rate $\lambda(t)$ is equal to the turnover rate $\tau(t)$. The likelihood of internode distances is directly obtained from Equation 1 by substitution.

Under models with varying diversity, the expected number of species at time t is obtained by solving the differential equation:

$$\frac{dN(t)}{dt} = \left[-\lambda(t) + \mu(t)\right]N(t)$$

and imposing the condition $N(t) = N_0$. Once this equation solved, the likelihood of internode distances is obtained from Equation 1 by substitution.

Under Model 3, the solution is $N(t) = N_0 e^{(-\lambda_0 + \mu_0)t}$.

Under Model 4a, $N(t) = N_0 \exp\left[\frac{\lambda_0}{\alpha}\left(1 - e^{\alpha t}\right) + \mu_0 t\right]$.

Under Model 4b, $N(t) = N_0 \exp\left[-\lambda_0 t - \frac{\mu_0}{\beta}\left(1 - e^{\beta t}\right)\right]$.

Under Model 4c, $N(t) = N_0 \exp\left[\frac{\lambda_0}{\alpha}(1 - \varepsilon)\left(1 - e^{\alpha t}\right)\right]$.

Under Model 4d, $N(t) = N_0 \exp\left[\frac{\lambda_0}{\alpha}\left(1 - e^{\alpha t}\right) - \frac{\mu_0}{\beta}\left(1 - e^{\beta t}\right)\right]$.

Under Model 5, $N(t) = N_0 e^{-\lambda_0 t}$.

Under Model 6, $N(t) = N_0 \exp\left[\frac{\lambda_0}{\alpha}\left(1 - e^{\alpha t}\right)\right]$.

Appendix 2. Power of the coalescent approach.

Figure S1. The coalescent approach provides a robust method for estimating rates from partially sampled phylogenies: case of constant diversity. Data points and error bars are the median and 95% quantile range of maximum likelihood rate estimates for 1000 phylogenies simulated under Model 2 (diversity is constant, with speciation events immediately following extinction events, and the rate of events decays exponentially through time). Dotted horizontal lines indicate the true parameter values. Clade size N_0=100.

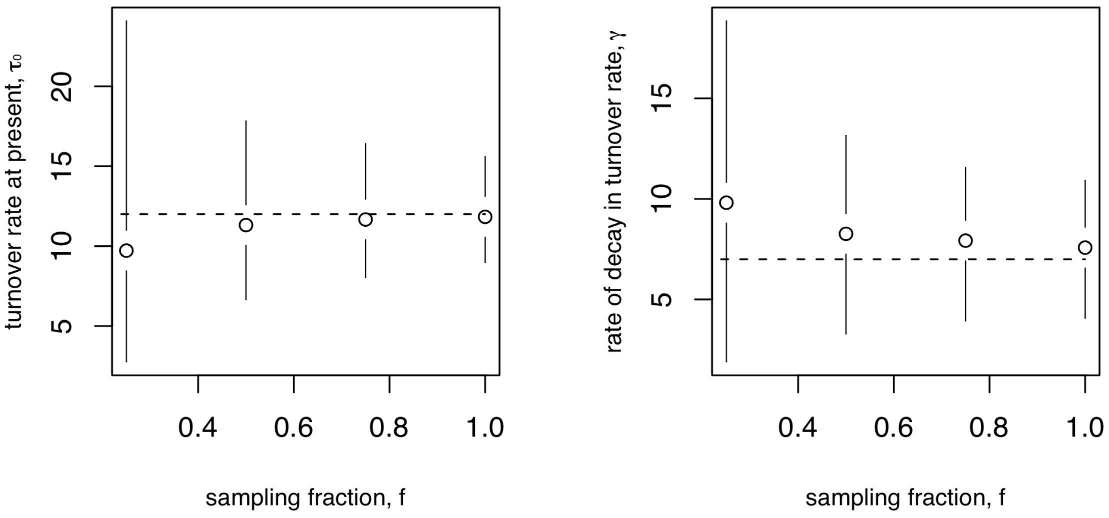

Figure S2. The coalescent approach allows us to select, among a set of models, the one that most likely gave rise to the phylogenetic branch lengths in an empirical phylogeny. The six phylogenies shown here are bird phylogenies from Phillimore & Price (2008) that exemplify a best-fit for each of six different models. For example, the *Tauraco* phylogeny, with short terminal branches, was consistent with a model of saturated diversity and constant turnover rate (Model 1); wherease the *Sylvia* phylogeny, with longer terminal branches, was consistent with a model of saturated diversity and decaying turnover rate (Model 2). In many cases, phylogenies have similar branch-length patterns (e.g. the *Sylvia* and *Sterna* phylogenies), and quantitative methods are required to assess their consistency with different diversification scenarios. For example, although they appear similar, the *Sylvia* phylogeny is most consistent with a scenario in which extinction events are as frequent as speciation events (Model 2), whereas the *Sterna* phylogeny is most consistent with a scenario without extinction (Model 6).

Tauraco, Model 1

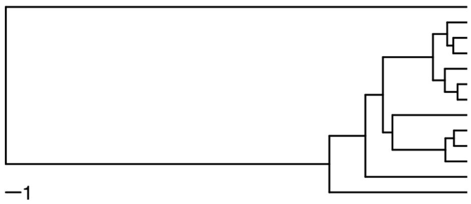

Sylvia, Model 2

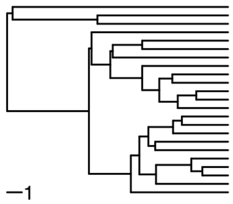

Albatross, Model 3

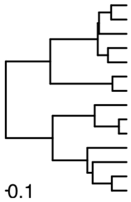

Anas, Model 4a

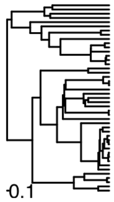

Myiarchus, Model 5

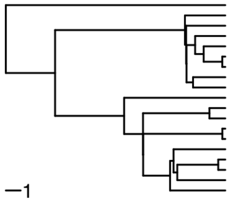

Sterna, Model 6

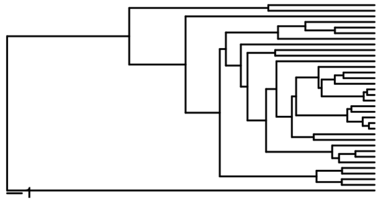

Figure S3. Our model selection procedure provides a robust method for testing diversity expansion and time variation in rates. In order to test the accuracy of our inferences, we simulated phylogenies with constant diversity and constant rates (100 phylogenies simulated under Model 1 with clade size N_0=100 and sampling fraction f = 0.75). As desired, the model selection procedure virtually always selected models with constant diversity, time-constant rates, and positive extinctions (red, blue, and green panels, respectively). Thus, the results in the main text regarding the prevalence of time-varying rates and expanding diversity for empirical phylogenies (Figure 4) are not artifacts of the coalescent approach or our procedure for model selection.

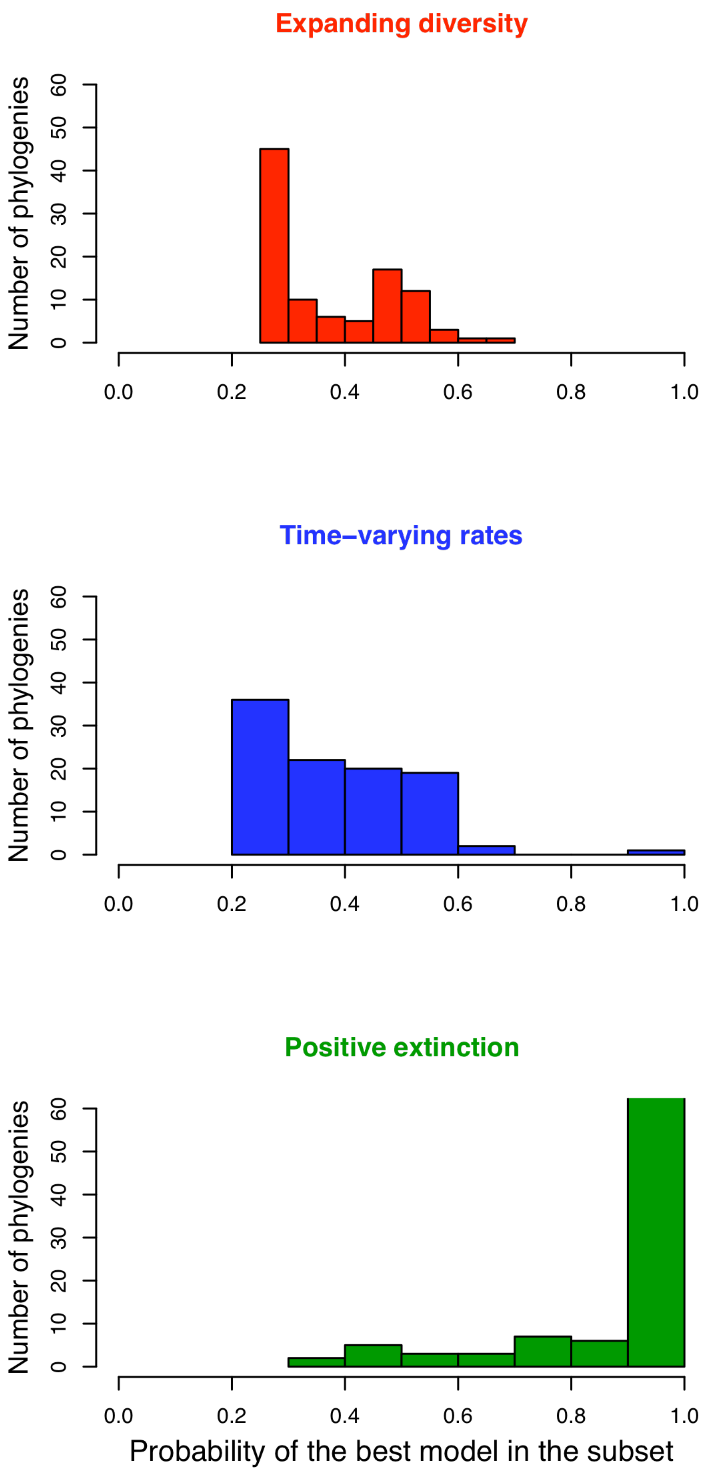

Expanding diversity
Number of phylogenies
0
10
20
30
40
50
60
0.0
0.2
0.4
0.6
0.8
1.0
Time−varying rates
Number of phylogenies
0
10
20
30
40
50
60
0.0
0.2
0.4
0.6
0.8
1.0
Positive extinction
Number of phylogenies
0
10
20
30
40
50
60
0.0
0.2
0.4
0.6
0.8
1.0
Probability of the best model in the subset

Appendix 3. Empirical results.

For Figure S4 through Figure S8, shown below, each histogram reports the distribution of phylogenies with respect to the relative probability of the best model in each subset of models. The results per phylum and restricted to the birds phylogenies from Phillimore & Price [1] are similar to those obtained for the full set of 289 phylogenies – i.e. rates generally vary through time, and diversity is still expanding at present.

Figure S4. Dynamics of diversification for the chordate phylogenies

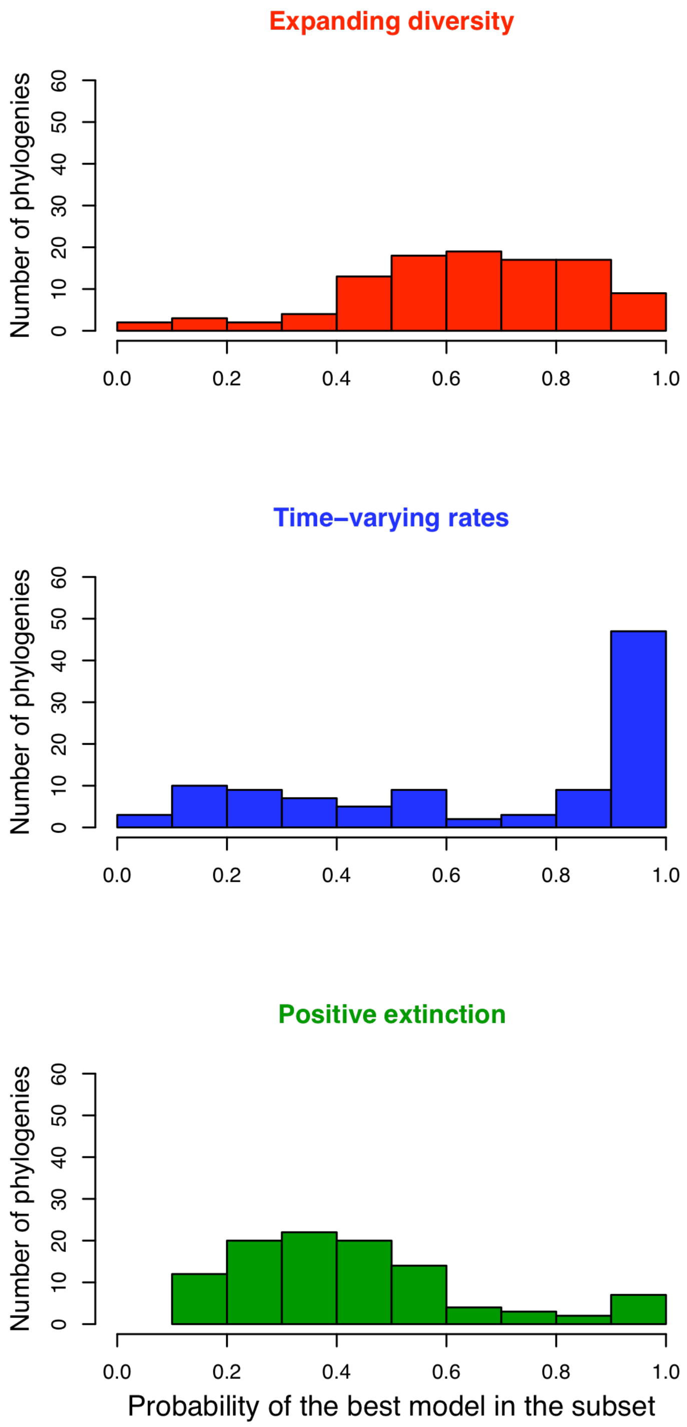

Figure S5. Dynamics of diversification for the mollusk phylogenies

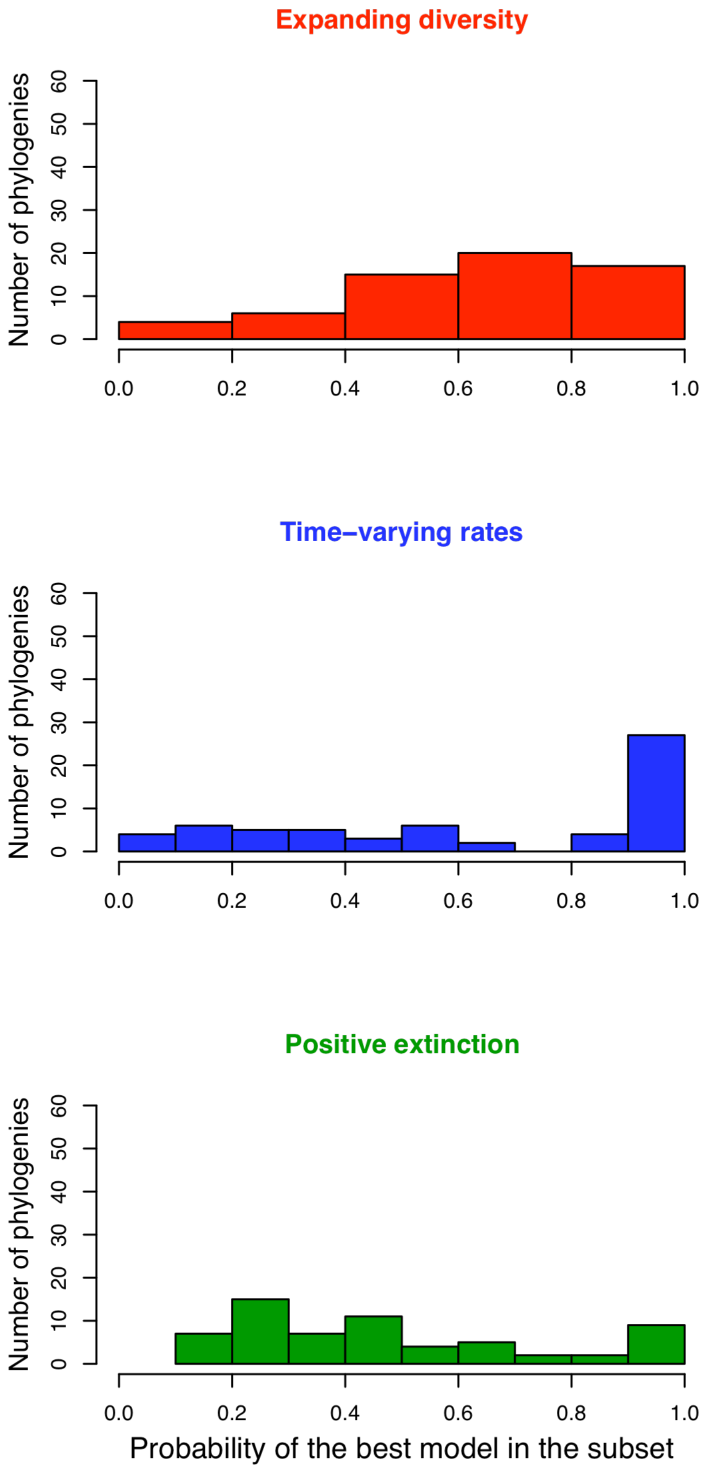

Figure S6. Dynamics of diversification for the arthropod phylogenies

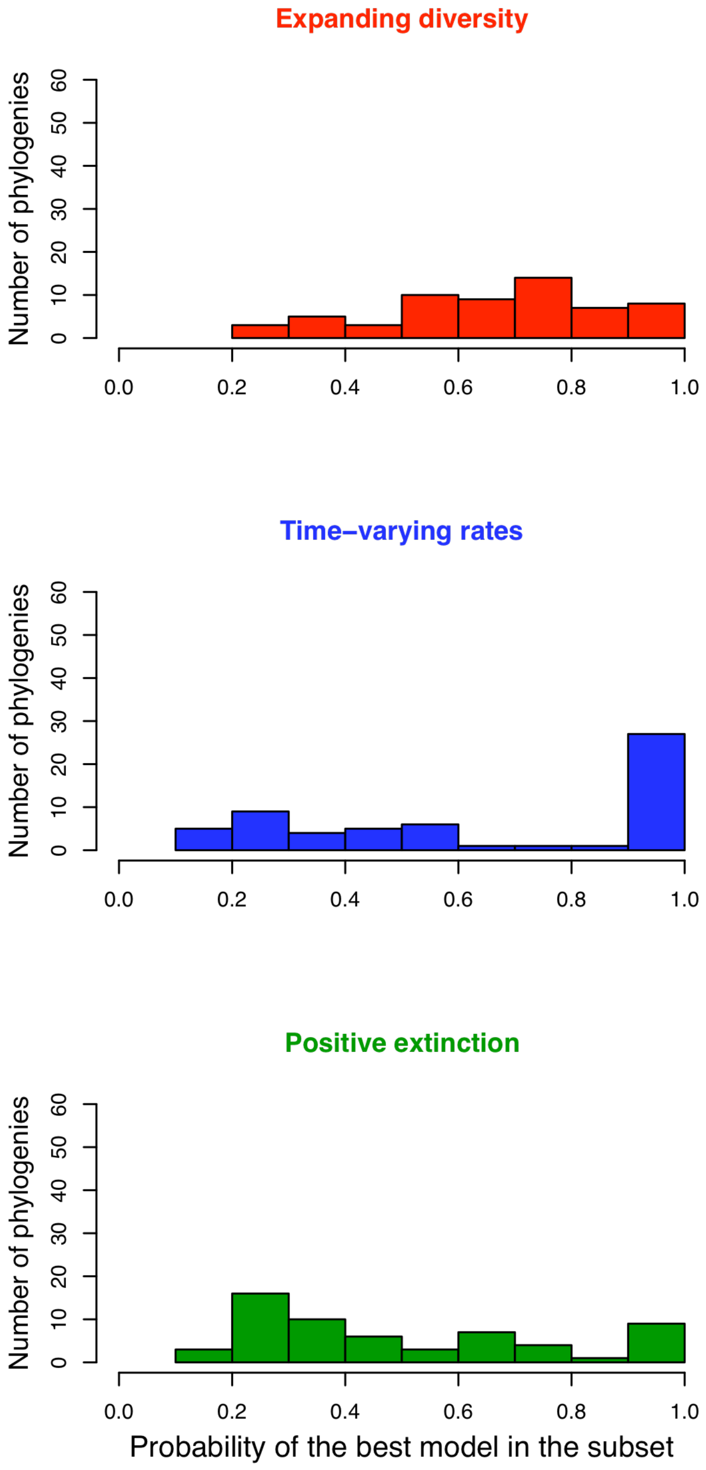

Figure S7. Dynamics of diversification for the magnoliophyte phylogenies

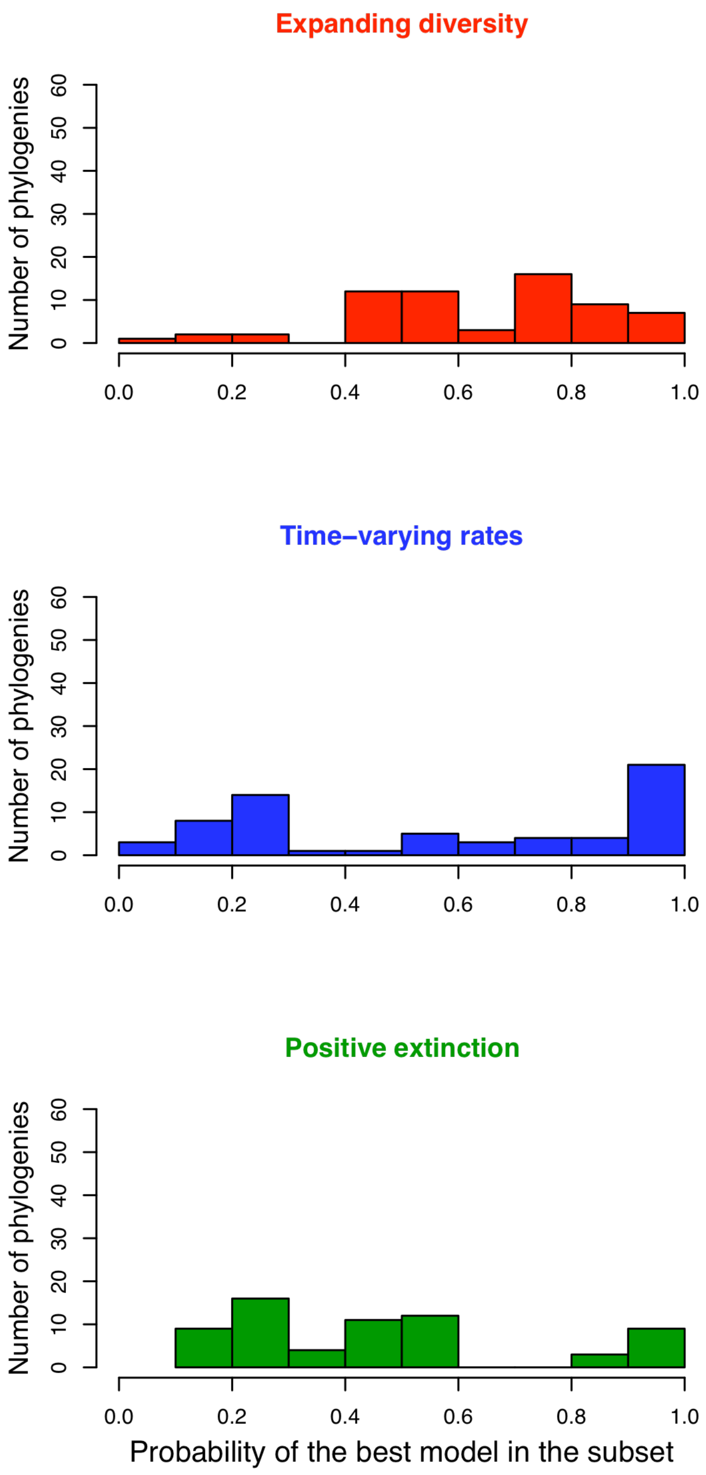

Figure S8. Dynamics of diversification across the bird phylogenies from Phillimore & Price [1].

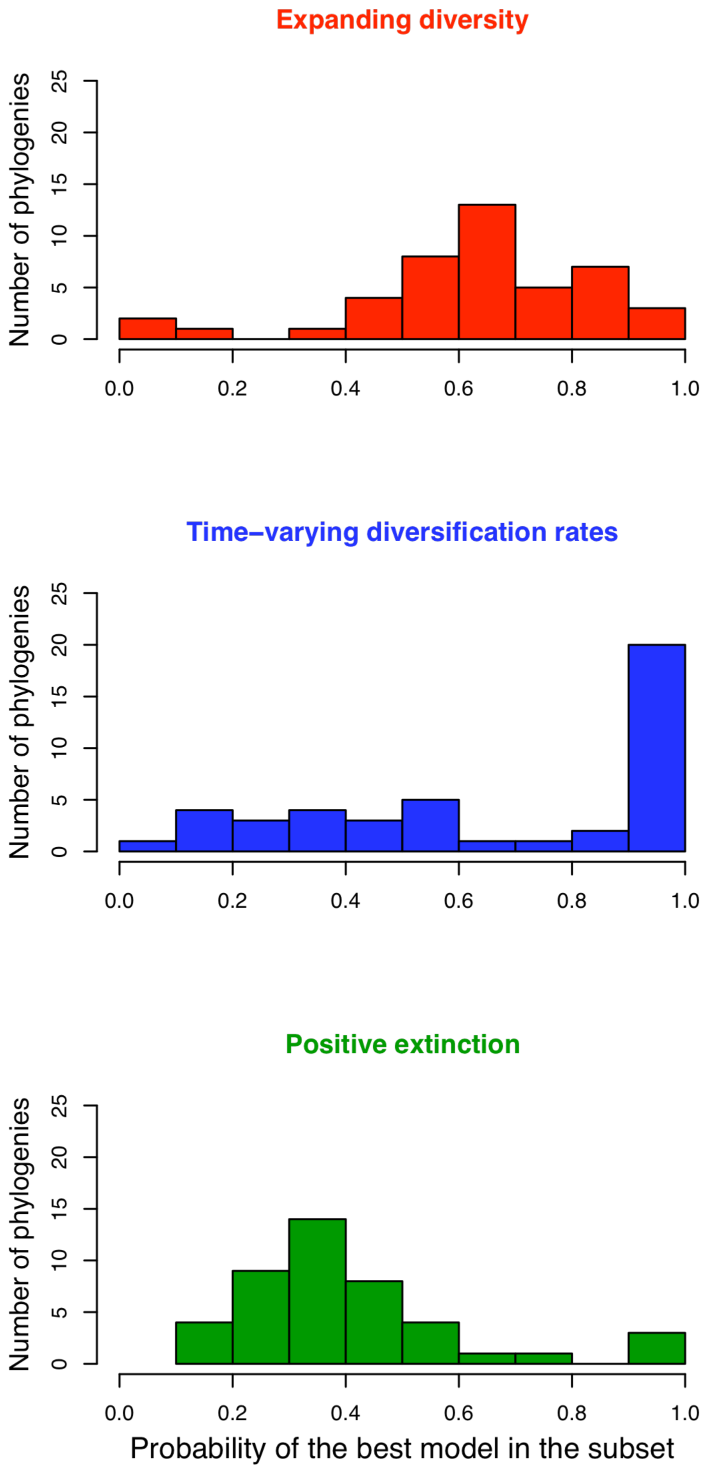

Figure S9. Robustness of the results to the fraction of species sampled and phylogenetic imbalance. Left column: effect of the fraction of species sampled; right column: effect of phylogenetic imbalance (measured as the tree-splitting parameter, [2]). In blue: chordata; in yellow: mollusca; in red: arthropoda; in green: magnoliophyta. The fraction of species sampled and phylogenetic imbalance had no effect on model selection, suggesting that the results were not biased by undersampling or inhomogeneous rate across lineages.

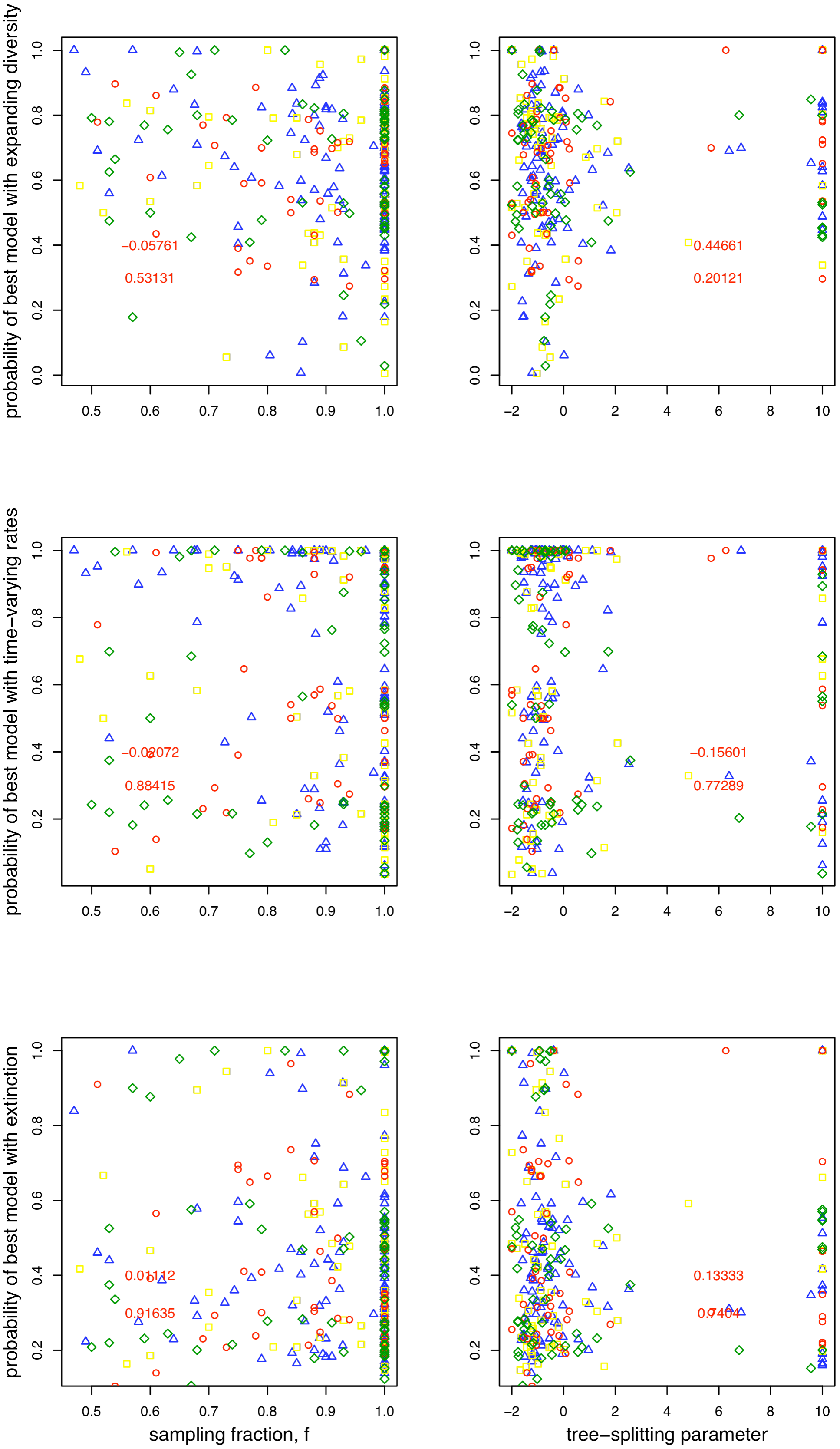

probability of best model with expanding diversity
probability of best model with time−varying rates
probability of best model with extinction
sampling fraction, f
tree−splitting parameter
−0.05761
0.53131
0.44661
0.20121
−0.02072
0.88415
−0.15601
0.77289
0.01112
0.91635
0.13333
0.7404

Figure S10. Robustness of the results to phylogeny size and clade size. Left column: effect of phylogeny size; right column: effect of clade size. In blue: chordata; in yellow: mollusca; in red: arthropoda; in green: magnoliophyta. The positive correlation of the relative probability of the best model with time-varying rates with phylogeny size and clade size (middle row) suggests that the few phylogenies consistent with the hypothesis that rates are constant through time are phylogenies with too little data to detect time-variation in rates. The positive correlation of the probability of the best model with positive extinction with phylogeny size and clade size (bottom row) suggests that the non-detection of extinctions in most phylogenies could be an artifact of analyzing small phylogenies. The negative correlation of the relative probability of the best model with expanding diversity with phylogeny size (top row, left panel) suggests that the result that most phylogenies are consistent with the expanding diversity hypothesis may be biased by the presence of many small phylogenies.

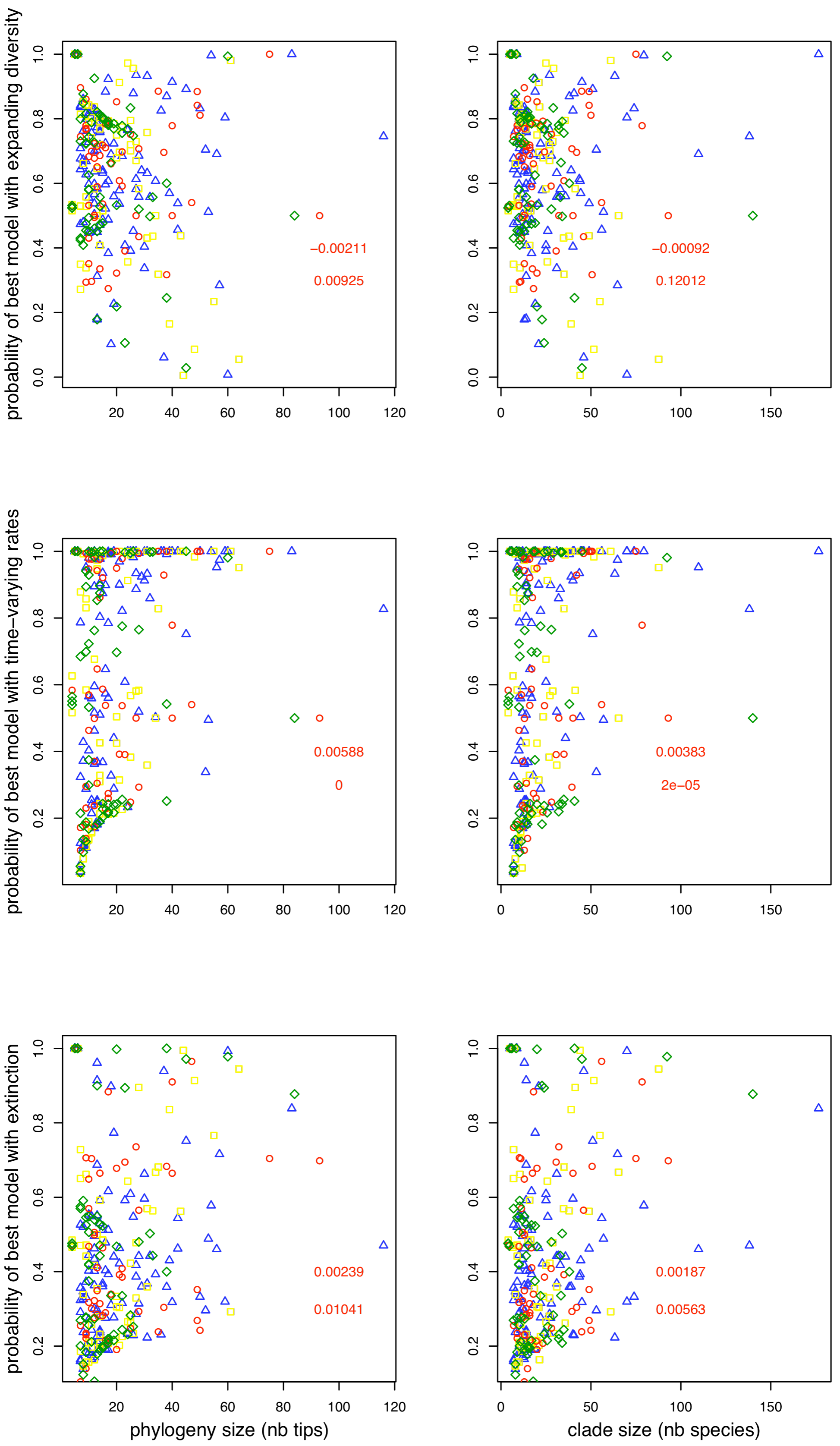
probability of best model with expanding diversity
-0.00211
0.00925
-0.00092
0.12012
probability of best model with time-varying rates
0.00588
0
0.00383
2e-05
probability of best model with extinction
0.00239
0.01041
0.00187
0.00563
phylogeny size (nb tips)
clade size (nb species)

Figure S11. Model fits for phylogenies with more than 10 tips (199 phylogenies). Each histogram reports the distribution of phylogenies with respect to the relative probability of the best model in each subset of models. The results are similar to those obtained for the 289 phylogenies – i.e. rates are generally time-varying, and diversity is still expanding at present.

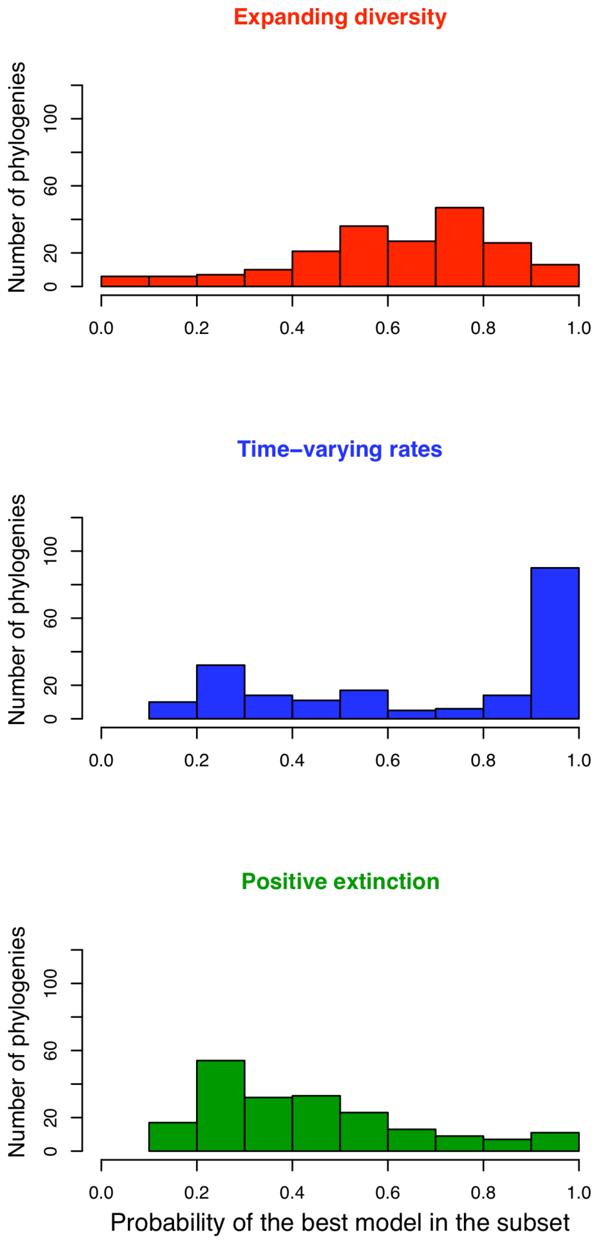

Figure S12. Model fits for phylogenies with more than 50 tips (16 phylogenies).

Each histogram reports the distribution of phylogenies with respect to the relative probability of the best model in each subset of models. The largest phylogenies strongly support the hypothesis that diversity is expanding with time-varying rates. Furthermore, they also detect significant extinctions.

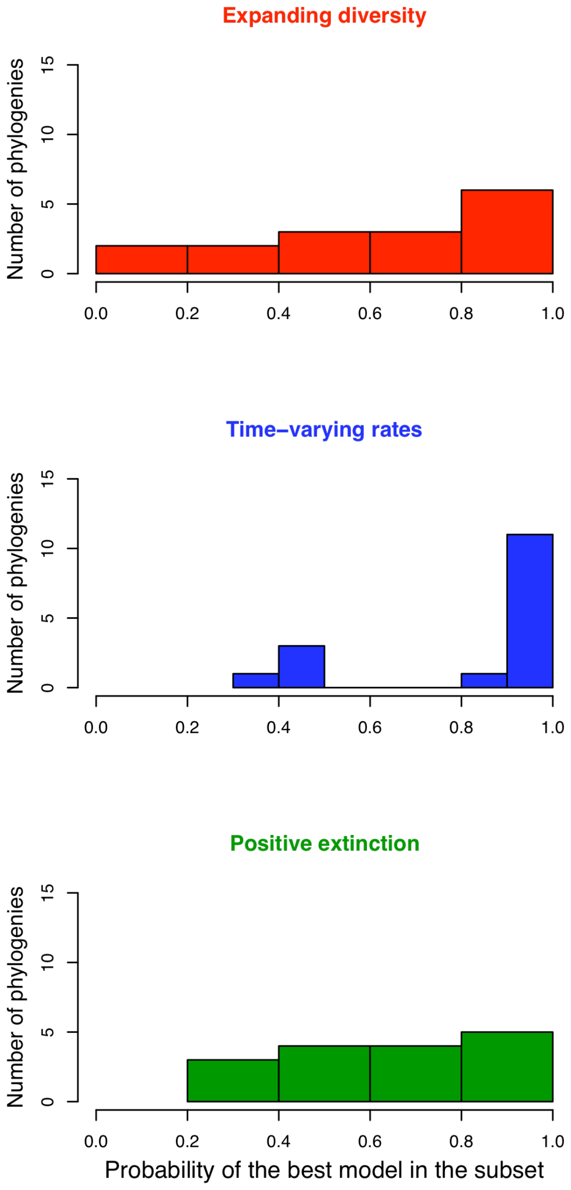

Appendix 4. Extinction rates inferred from molecular phylogenies

Figure S13. Comparison between extinction rates estimated using the forward-time approach and estimated using the coalescent approach. The histograms report the number of phylogenies with a given estimated extinction fraction at present, for those phylogenies best-fit by Model 4a. Left column: rates estimated using the SPVAR model from Rabosky & Lovette [3,4]; Right column: rates estimated using the coalescent approach. Many phylogenies for which the coalescent approach infers a high extinction fraction have an estimated fraction close to zero when inferred by the forward-time approach. The same qualitative pattern holds when considering only those phylogenies in which the number of species sampled exceeded 80% (bottom panels).

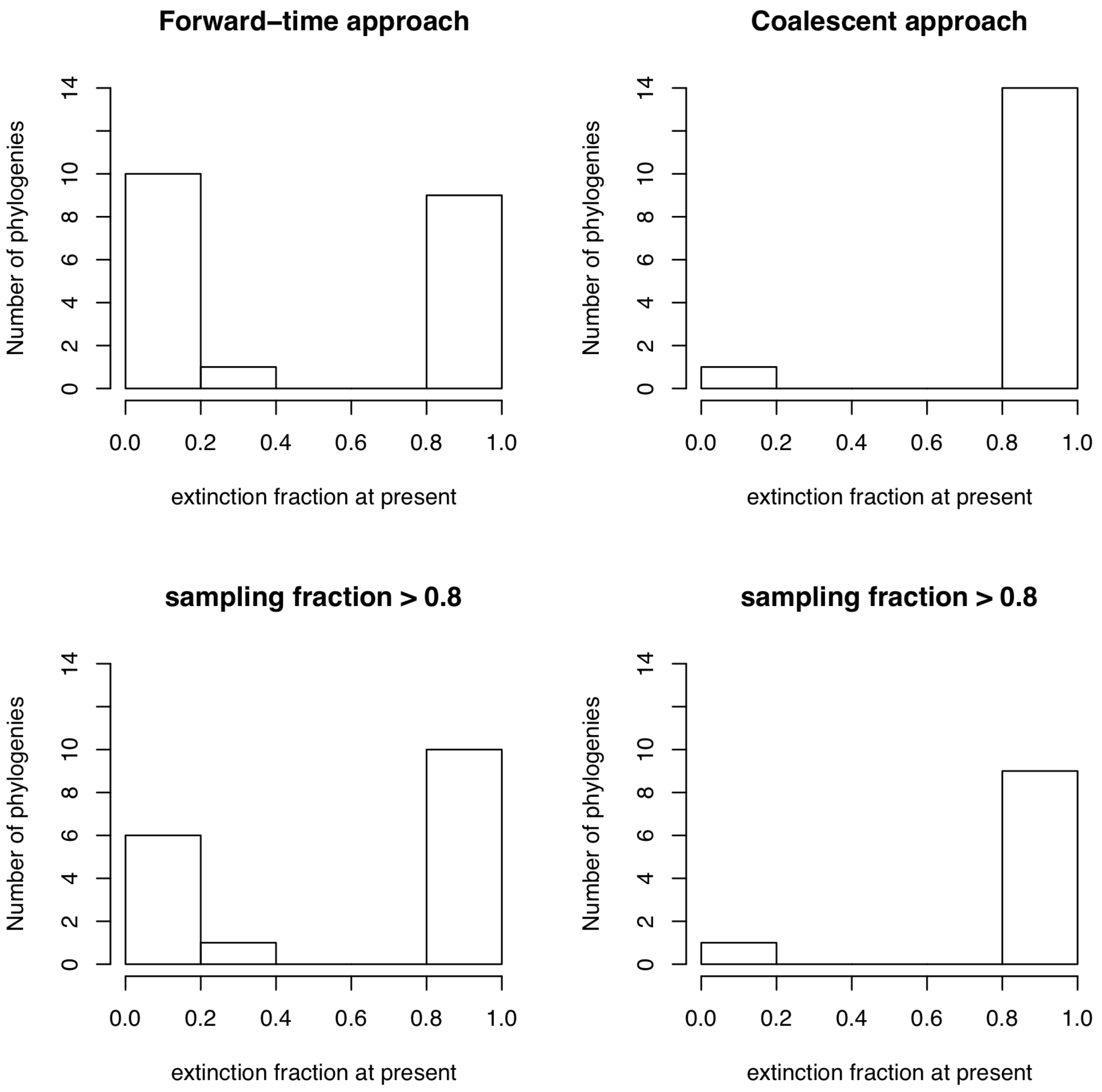

Forward–time approach
Coalescent approach
sampling fraction > 0.8
sampling fraction > 0.8
Number of phylogenies
extinction fraction at present
0.0 0.2 0.4 0.6 0.8 1.0
0 2 4 6 8 10 14

Table S1. Parameter estimates for the 10 phylogenies with more than 50 tips displaying positive extinction.

Phylogeny	Phylogeny size (# tips)	Best-fit model	Model properties	Parameters		Parameter estimate (Myr^{-1})
Cephaloleia (beetles) [5]	75	Model 4a	expanding diversity<br>time-varying rates<br>positive extinction	λ_0<br>α<br>μ_0	speciation rate at present<br>exponential variation in speciation rate<br>extinction rate	0.059<br>0.18<br>0.059
Leucadendron (flowering plants) [6]	60	Model 4a	expanding diversity<br>time-varying rates<br>positive extinction	λ_0<br>α<br>μ_0	speciation rate at present<br>exponential variation in speciation rate<br>extinction rate	1<br>0.34<br>1
Rana (frogs) [7]	57	Model 2	constant diversity<br>time-varying rates<br>positive extinction	τ_0<br>γ	turnover rate at present<br>exponential variation in turnover rate	0.16<br>0.47
Bursera (flowering plants) [8]	64	Model 2	constant diversity<br>time-varying rates<br>positive extinction	τ_0<br>γ	turnover rate at present<br>exponential variation in turnover rate	0.11<br>0.26
Bicyclus (butterflies) [9]	54	Model 4a	expanding diversity<br>time-varying rates<br>positive extinction	λ_0<br>α<br>μ_0	speciation rate at present<br>exponential variation in speciation rate<br>extinction rate	0.04<br>0.07<br>0.04
Bolitoglossa (salamenders) [10]	55	Model 2	constant diversity<br>time-varying rates<br>positive extinction	τ_0<br>γ	turnover rate at present<br>exponential variation in turnover rate	0.04<br>0.33
Tylomelania (snails) [11]	93	Model 3	expanding diversity<br>time-constant rates<br>positive extinction	λ_0<br>μ_0	speciation rate<br>extinction rate	2.53<br>1.12
Cephaloleia (beetles) [12]	83	Model 4a	expanding diversity<br>time-varying rates<br>positive extinction	λ_0<br>α<br>μ_0	speciation rate at present<br>exponential variation in speciation rate<br>extinction rate	0.03<br>0.032<br>0.03
Streptocarpus (flowering plants) [13]	84	Model 2	constant diversity<br>time-varying rates<br>positive extinction	τ_0<br>γ	turnover rate at present<br>exponential variation in turnover rate	0.38<br>0.12
Turdus (birds) [1]	60	Model 2	constant diversity<br>time-varying rates<br>positive extinction	τ_0<br>γ	turnover rate at present<br>exponential variation in turnover rate	0.06<br>0.53

Appendix 5. Branch lengths and phylogenetic imbalance of best-fit models

Figure S14. The best-fit model accurately reproduces the observed phylogenetic branch-lengths, but it does not accurately reproduce phylogenetic imbalance. The best-fit model typically underestimates phylogenetic imbalance (top panel), but produces accurate gamma values (bottom panel). The *y* coordinate corresponds to the mean of the tree-splitting parameters and gamma values for 100 phylogenies simulated under the best-fit model.

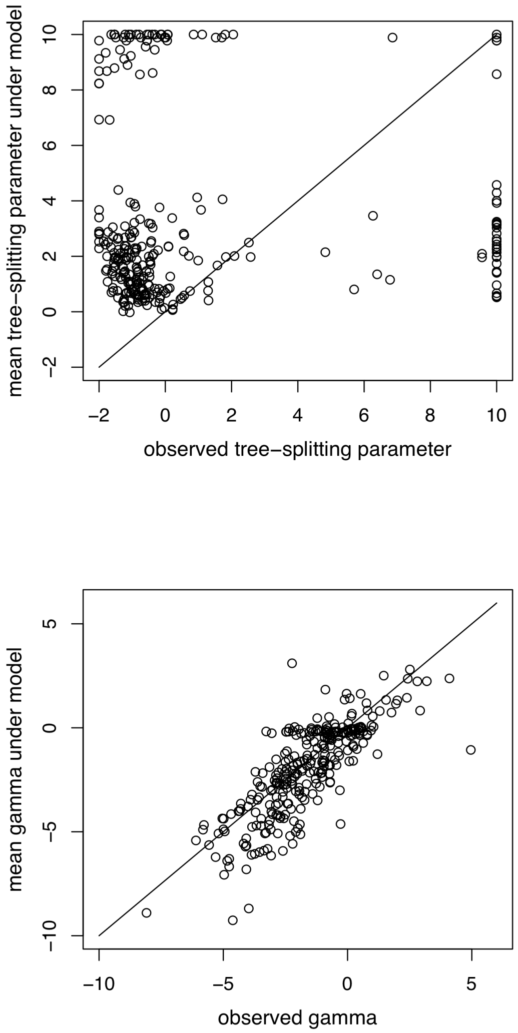

Supplementary References

1. Phillimore AB, Price TD (2008) Density-dependent cladogenesis in birds. PLoS Biol 6: e71.

2. Blum MGB, Francois O (2006) Which random processes describe the tree of life? A large-scale study of phylogenetic tree imbalance. Syst Biol 55: 685-691.

3. Rabosky DL (2006) LASER: a maximum likelihood toolkit for detecting temporal shifts in diversification rates from molecular phylogenies. Evol Bioinform 2: 273.

4. Rabosky DL, Lovette IJ (2008) Explosive evolutionary radiations: decreasing speciation or increasing extinction through time? Evolution 62: 1866-1875.

5. McKenna DD, Farrell BD (2005) Molecular phylogenetics and evolution of host plant use in the Neotropical rolled leaf hispine beetle genus Cephaloleia (Chevrolat)(Chrysomelidae: Cassidinae). Mol Phylo Evol 37: 117-131.

6. Barker NP, Vanderpoorten A, Morton CM, Rourke JP (2004) Phylogeny, biogeography, and the evolution of life-history traits in Leucadendron (Proteaceae). Mol Phylo Evol 33: 845-860.

7. Hillis DM, Wilcox TP (2005) Phylogeny of the New World true frogs (Rana). Mol Phyl Evol 34: 299-314.

8. Becerra JX (2003) Evolution of Mexican Bursera (Burseraceae) inferred from ITS, ETS, and 5S nuclear ribosomal DNA sequences. Mol Phyl Evol 26: 300-309.

9. Monteiro A, Pierce NE (2001) Phylogeny of Bicyclus (Lepidoptera: Nymphalidae) Inferred from COI, COII, and EF-1 [alpha] Gene Sequences. Mol Phyl Evol 18: 264-281.

10. Parra-Olea G, GarcÌa-ParÌs M, Wake DB (2004) Molecular diversification of salamanders of the tropical American genus Bolitoglossa (Caudata: Plethodontidae) and its evolutionary and biogeographical implications. Biol J Linn Soc 81.

11. Rintelen T, Wilson AB, Meyer A, Glaubrecht M (2004) Escalation and trophic specialization drive adaptive radiation of freshwater gastropods in ancient lakes on Sulawesi, Indonesia. Proc R Soc Lond B 271: 2541-2549.

12. McKenna DD, Farrell BD (2006) Tropical forests are both evolutionary cradles and museums of leaf beetle diversity. Proc Natl Acad Sci USA 103: 10947–10951.

13. Moller M, Cronk QCB (2001) Evolution of morphological novelty: a phylogenetic analysis of growth patterns in Streptocarpus (Gesneriaceae). Evolution 55: 918-929.
